# Biologically Mediated Nanoparticle Synthesis as a Potential Green Strategy: Principles, Methods, and Pharmaceutical Applications

**DOI:** 10.3390/pharmaceutics18091151

**Published:** 2026-09-14

**Authors:** Mahmoud M. Mokhtar, Rahma Wael Soliman, Jassy Salim, Shrouk Khaled Mohamed, Lobna Abdelsalam, Rehab Abdelmonem

**Affiliations:** 1 Faculty of Pharmacy, Misr University for Science and Technology, 6th of October City 12566, Egypt; 2 Faculty of Pharmacy, Helwan University, Helwan, Cairo 11795, Egypt; rahma.2025soliman@pharm.helwan.edu.eg; 3 Faculty of Medicine, Modern University for Technology and Information, Mokattam, Cairo 11571, Egypt; 4 Kasr Al-Ainy Faculty of Medicine, Cairo University, Cairo 11956, Egypt; shorouk.khaled2023@students.kasralainy.edu.eg; 5 Faculty of Pharmacy and Biotechnology, German University in Cairo (GUC), New Cairo 11835, Egypt; lobna.elansary31@gmail.com; 6 Industrial Pharmacy Department, College of Pharmaceutical Sciences and Drug Manufacturing, Misr University for Science and Technology (MUST), 6th of October City 12566, Egypt

**Keywords:** green synthesis, nanoparticles, biological synthesis, plant-mediated synthesis, microbial synthesis, nanomedicine, nanoparticle characterization, sustainable nanotechnology

## Abstract

Nanoparticles have gained considerable attention as important platforms in pharmaceutical and biomedical research due to their size, morphology, surface chemistry, and colloidal behavior, which can affect drug delivery, antimicrobial activity, diagnostics, imaging, and therapeutic performance. However, conventional chemical and physical synthesis routes may require hazardous reagents, high energy demands, and procedures that raise environmental, safety, and scalability concerns. In this context, biologically mediated synthesis has emerged as an approach in which plants, microorganisms, including fungi and algae, and isolated biomolecules contribute to nanoparticle formation by acting as reducing, capping, and stabilizing agents. This narrative review evaluates these routes as potential green strategies, covering formation principles, biological sources, nanoparticle classes, characterization challenges, pharmaceutical applications, comparison with conventional synthesis, and limitations. The reviewed evidence indicates that the composition of biological sources and reaction conditions can shape nanoparticle size, morphology, surface chemistry, stability, and biological activity, highlighting green synthesis as a design-dependent process rather than a simple substitute for chemical reducing agents. Integrated characterization using optical, spectroscopic, diffraction, microscopic, and colloidal techniques is essential for interpreting nanoparticle identity and reproducibility. Biologically mediated nanoparticles show experimental and preclinical potential in drug delivery, antimicrobial therapy, anticancer research, biosensing, imaging, and diagnostics; however, most evidence remains preclinical and model-dependent. Translation remains limited by source variability, batch inconsistency, scale-up and purification challenges, stability, incomplete safety evidence, and regulatory uncertainty. Progress will require standardized process control, comparative life cycle and techno-economic assessment, scalable manufacturing, and rigorous pharmacokinetic, biodistribution, toxicological, and regulatory validation.

## 1. Introduction

Nanoparticles (NPs) are generally defined as particles within the 1–100 nm size range, and at this scale, they exhibit unique physicochemical properties compared with larger-scale materials, including high surface-area-related reactivity as well as altered optical, electronic, magnetic, thermal, mechanical, and catalytic behavior [1,2,3,4,5,6]. Nanoparticle size, shape, and surface characteristics are major determinants of biological behavior by influencing cellular interactions, tissue distribution, and systemic fate [7]. Nanotechnology has become increasingly important in biomedicine because it enables advanced platforms for drug delivery, diagnostics, and imaging [8]. In drug delivery, nanocarriers can improve controlled release, bioavailability, and tissue targeting of therapeutic agents [9,10]. In diagnostics, nanoparticle-based systems can enhance analytical sensitivity and support rapid detection [11,12], whereas in biomedical imaging, they can function as targeted contrast platforms that improve lesion detection [13]. These properties are important in pharmaceutical formulation research because nanoparticle design can strongly affect drug solubility, release behavior, targeting efficiency, physicochemical stability, and toxicity [14,15,16].

Conventional nanoparticle synthesis is broadly categorized into chemical, physical, and biological approaches, with chemical and physical methods representing the traditional routes [17]. Some chemical synthesis routes may involve hazardous reagents, which pose toxicity, handling, and waste-disposal concerns [18,19]. Some physical methods require high temperatures, elevated pressure, or other energy-intensive conditions, making these approaches costly, time-consuming, and less suitable for sustainable large-scale production [20,21]. As a result, these limitations in some conventional processes contribute to broader environmental and safety concerns and are operationally less compatible with sustainable and scalable production, which has encouraged growing interest in greener synthesis strategies [5,17].

Green synthesis refers to the production of materials, particularly nanoparticles, through environmentally compatible and sustainable processes that reduce hazardous reagent use, minimize waste, and employ safer or renewable biological resources [22,23]. Biologically mediated nanoparticle synthesis uses biological systems or naturally derived biomolecules as reducing and stabilizing agents, thereby reducing reliance on the use of hazardous chemicals [24,25,26]. As a result, green synthesis has attracted increasing attention because it may offer advantages such as lower toxicity, improved biocompatibility, cost-effectiveness, and sustainability [26,27].

Biological systems can mediate nanoparticle synthesis by providing naturally occurring reducing and stabilizing components. Plant extracts are widely used because their phytoconstituents serve as reducing, capping, and stabilizing agents, whereas microbial systems, particularly fungi, function as efficient biocatalytic platforms for nanoparticle formation [25,28,29,30]. Biomolecule-mediated synthesis is a distinct approach in which isolated or purified biomolecules, including proteins, enzymes, polysaccharides, and nucleotides, are used as defined reducing, templating, or capping agents rather than complex whole-extract systems [31,32,33,34]. This approach may offer greater compositional control than whole-extract systems because the reducing or capping molecules are more clearly defined. Biomolecules such as polyphenols, terpenoids, polysaccharides, proteins, and enzymes are key mediators of these processes, supporting the use of plants, microorganisms, and their derived biomolecules in biologically mediated nanoparticle synthesis [25,31,32,33,35].

Despite the growing body of evidence on biologically mediated nanoparticle synthesis, the field remains fragmented across biological sources, nanoparticle classes, and experimental applications. Many studies focus on specific biological sources, nanoparticle types, or biomedical applications rather than considering the main synthesis principles, methods, characterization techniques, pharmaceutical relevance, and limitations in one clear framework. Although biologically mediated synthesis can reduce chemical hazards, its pharmaceutical translation remains limited by biological-source variability, batch-to-batch inconsistency, scale-up challenges, and the need for standardized characterization and safety validation.

This review evaluates biologically mediated nanoparticle synthesis as a potential green strategy, focusing on biological synthesis principles; plant-, microorganism-, and biomolecule-mediated routes; major nanoparticle classes; biological surface and capping effects; characterization challenges; pharmaceutical applications; and translational limitations. Biological mediation is not treated as sufficient evidence that the overall production process is environmentally green.

## 2. Review Scope and Literature Selection

This narrative review examines biologically mediated nanoparticle synthesis as a potential green strategy, focusing on the principles of nanoparticle formation, plant-, microorganism-, and biomolecule-mediated synthesis routes, challenges in characterizing biological capping layers, major nanoparticle classes, and pharmaceutical applications. The evidence discussed the potential pharmaceutical use of green-synthesized nanoparticles in drug delivery, antimicrobial therapy, anticancer applications, and wound healing. Studies addressing only environmental remediation, wastewater treatment, agriculture, photocatalysis, or non-pharmaceutical industrial applications were considered outside the scope of the revised review.

The literature search was conducted in PubMed from 30 April to 20 July 2026. The search combined terms relating to “green synthesis”, “biological synthesis”, “nanoparticles”, “plant-mediated synthesis”, “microbial synthesis”, “biomolecule-mediated synthesis”, “characterization”, “drug delivery”, “antimicrobial activity”, “anticancer activity”, and “pharmaceutical applications”.

The review focused primarily on English-language publications from 2021 to 2026. Primary experimental studies were prioritized when discussing synthesis procedures, nanoparticle characterization, biological activity, pharmaceutical applications, and translational limitations. Review articles were used mainly to establish broader concepts. Publications were excluded when they lacked direct relevance to biologically mediated synthesis or addressed only non-pharmaceutical applications.

We selected the literature based on its relevance to the synthesis route, nanoparticle type, characterization quality, experimental model, pharmaceutical application, and translational limitations. The selected evidence was synthesized narratively rather than through meta-analysis, with comparisons organized around biological synthesis routes, nanoparticle characteristics, pharmaceutical relevance, reproducibility, safety, and scalability. Because this was a narrative review, no formal systematic-review risk-of-bias assessment was performed.

## 3. Principles of Biologically Mediated Nanoparticle Synthesis

The principles of biologically mediated nanoparticle synthesis are based on the interaction between biological reducing, capping, and stabilizing systems under controllable reaction conditions. Biologically mediated synthesis is presented as a multivariable process involving metal-ion reduction, nucleation, particle growth, surface capping, and colloidal stabilization.

### 3.1. Biological Reduction Mechanism

Mechanistically, biologically mediated synthesis can be understood as a coordinated sequence in which biological metabolites or enzymes reduce metal ions, initiate nucleation, regulate particle growth, and subsequently adsorb onto the nanoparticle surface as capping/stabilizing layers that influence size, morphology, surface charge, and colloidal stability [36,37]. “Capping” refers to biomolecular adsorption that controls nanoparticle growth and surface properties, whereas “stabilization” refers to the prevention of aggregation in colloidal suspension; a biomolecular surface layer can contribute to both functions [38,39]. Plant extracts contain phytochemicals such as phenolic compounds, flavonoids, tannins, and saponins, which can act as reducing agents during biologically mediated synthesis [40]. For example, in silver nanoparticle synthesis, electron-donating phytochemicals can donate electrons to silver ions, converting Ag^+^ into elemental silver Ag^0^, followed by nucleation and particle formation [41,42,43].

Proteins may also participate directly in metal-ion reduction in enzyme-mediated systems; for example, enzyme–metal nanohybrid synthesis showed that specific amino-acid sequences in lipase structure contributed to the reduction of metal ions into dispersed nanoparticles [44]. Similarly, microbial extracellular filtrates can support AgNP formation through nitrate reductase activity and nitrogen-containing biomolecules, which also help cap and stabilize the particles [45]. Surface-associated proteins were detected on actinobacteria-derived AgNPs, consistent with protein-mediated capping; the resulting nanoparticles also showed colloidal stability [46]. Optimization studies using bacterial supernatants and intracellular extracts showed that AgNP formation was influenced by the bacterial source, extract type, pH, and temperature. More specifically, nitrate reductase from *Aspergillus terreus* N4 has been reported to reduce Ag^+^ to Ag^0^ during AgNP biosynthesis, providing direct enzymatic evidence for the reduction step [47]. After reduction, phytochemicals may bind to the nanoparticle surface and function as natural capping agents through hydrogen bonding and electrostatic attraction [40]. FTIR-based studies further support that phenolic groups, proteins, and polysaccharides may remain associated with nanoparticle surfaces and contribute to capping and stabilization [48,49,50,51,52]. These biomolecular coatings help limit uncontrolled particle growth and aggregation, thereby influencing nanoparticle size, morphology, and colloidal stability [40]. Green-synthesized nanoparticles may show improved dispersion or stability when biological molecules participate in reduction, surface capping, or stabilization; however, these effects depend on the biological source, reaction conditions, and nanoparticle composition [40,53]. Green-synthesized AgNPs may exhibit sustained stability when plant-derived biomolecules participate in reduction and surface stabilization, whereas polysaccharide-polyphenol coatings improve SeNP stability across storage, pH, and temperature conditions [40,53]. Epigallocatechin gallate (EGCG) represents a polyphenolic stabilizer that can improve selenium nanoparticle dispersion, since SeNPs are prone to aggregation because of their high surface energy [53]. In starch microgel–EGCG selenium nanoparticles, EGCG improved dispersion and storage stability compared with uncoated or starch microgel-coated SeNPs [53]. Selected biologically capped formulations also showed negative zeta potentials and relatively narrow hydrodynamic size distributions, consistent with limited aggregation and colloidal stability [48,54,55,56,57]. AgNP formation may be initially indicated by a reddish-brown color change associated with surface plasmon resonance and assessed by UV–Vis spectroscopy [40]. Because reduction, nucleation, growth, and capping overlap during green synthesis, nanoparticle formation is sensitive to reaction conditions. Parameters including pH, temperature, precursor concentration, reaction time, and biological-extract concentration can influence nanoparticle yield, size distribution, morphology, and colloidal stability [58,59].

### 3.2. Factors Affecting Biologically Mediated Synthesis

#### 3.2.1. pH

pH is a critical reaction parameter in biologically mediated synthesis because it affects biomolecule ionization, metal-ion reduction, nucleation, particle growth, and colloidal stability. Experimental AgNP studies show that changing pH alters UV–Vis absorbance and SPR band intensity, reduction efficiency, hydrodynamic size, morphology, zeta potential, and aggregation behavior. In many AgNP systems, mild alkaline conditions tend to produce smaller and more stable nanoparticles, whereas strongly acidic or highly alkaline conditions may lead to irregular morphology or particle aggregation [60,61,62,63].

#### 3.2.2. Temperature

Temperature modulates reduction kinetics, nucleation, and particle growth. In the *Magnolia alba* AgNP study, nanoparticle formation was influenced by heating conditions, with optimized synthesis reported at 95 °C for 30 min in the Magnolia alba AgNP system [40]. Similarly, other optimization studies show that reaction temperature can influence AgNP formation, as indicated by changes in SPR absorbance intensity, synthesis efficiency, particle size, and dispersion-related properties [58,64,65]. Temperature can also influence particle size and dispersion behavior, but direct evidence for temperature-driven aggregation is less consistent; therefore, aggregation should be described cautiously as a possible outcome of non-optimal thermal conditions rather than a universal effect [64,66,67].

#### 3.2.3. Concentration of Metal Ions

Metal-ion precursor concentration affects the balance between nucleation and particle growth during biologically mediated synthesis by determining the availability of reducible ions relative to biological reducing and capping molecules [58,68]. Comparative optimization studies show that increasing AgNO_3_ concentration can increase SPR absorbance; however, when precursor levels exceed the available reducing and capping capacity, particle size and agglomeration may increase [64,69,70].

#### 3.2.4. Reaction Time

Reaction time affects metal-ion reduction, nucleation, and subsequent particle growth. In plant-mediated AgNP synthesis, progressive color changes may indicate increasing nanoparticle formation over time [40]. Reaction time should be controlled because time-course and optimization studies show that AgNP formation changes with incubation duration, as reflected by the development of the UV–Vis SPR band and optimized absorbance or formation output [58,64]. Longer or non-optimal reaction times may also increase particle size and PDI and alter size distribution or aggregation behavior. For example, prolonged synthesis increased AgNP size in both *Acacia cyanophylla* extract- and glutathione-mediated systems. However, changes in PDI and colloidal stability were system-dependent; therefore, reaction-time–driven aggregation should be described as a possible risk during overgrowth or insufficient stabilization rather than a fixed outcome [71,72].

#### 3.2.5. Plant Extract Concentration

Plant extract concentration determines the availability of plant-derived reducing and capping constituents during biologically mediated synthesis. Insufficient extract can limit AgNP formation, whereas higher extract concentrations can alter particle growth, size distribution, and morphology depending on the synthesis system [73]. Plant extract concentration should therefore be optimized rather than simply increased: experimental studies that varied extract concentration showed concentration-dependent changes in SPR absorbance, nanoparticle formation output, particle size, and aggregation behavior [74,75]. However, an unsuitable extract-to-metal ratio can lead to a wider particle-size distribution or lower dispersion stability. Therefore, extract concentration should be assessed alongside UV–Vis intensity, DLS/PDI, zeta potential, and microscopic morphology [74,76]. Overall, green nanoparticle synthesis should not be treated as a simple replacement of chemical reducers with biological extracts. It is a multivariable synthesis system in which biological source composition and reaction parameters together determine reduction efficiency, nucleation rate, growth behavior, surface chemistry, and colloidal stability. Therefore, reproducible biologically mediated synthesis requires systematic optimization and characterization rather than relying only on visible color change or apparent particle formation.

From a pharmaceutical quality-by-design perspective, biologically mediated nanoparticle production can be structured in terms of critical material attributes (CMAs), critical process parameters (CPPs), and critical quality attributes (CQAs). Biological-source composition, precursor characteristics, and extract or biomolecule concentration can function as CMAs, whereas pH, temperature, reaction time, precursor-to-biological-material ratio, and other operating conditions can act as CPPs when variation in these parameters alters nanoparticle quality. Experimental optimization studies of plant- and microorganism-mediated synthesis demonstrate that these variables can influence particle size, morphology, size distribution, surface charge, crystallinity, and colloidal stability [37,58,77]. These properties can therefore be considered relevant CQAs for biologically mediated nanoparticle systems, although their criticality and acceptable ranges must be established for the intended formulation rather than assumed to be universal.

## 4. Biological Sources for Nanoparticle Synthesis

**Figure 1:**
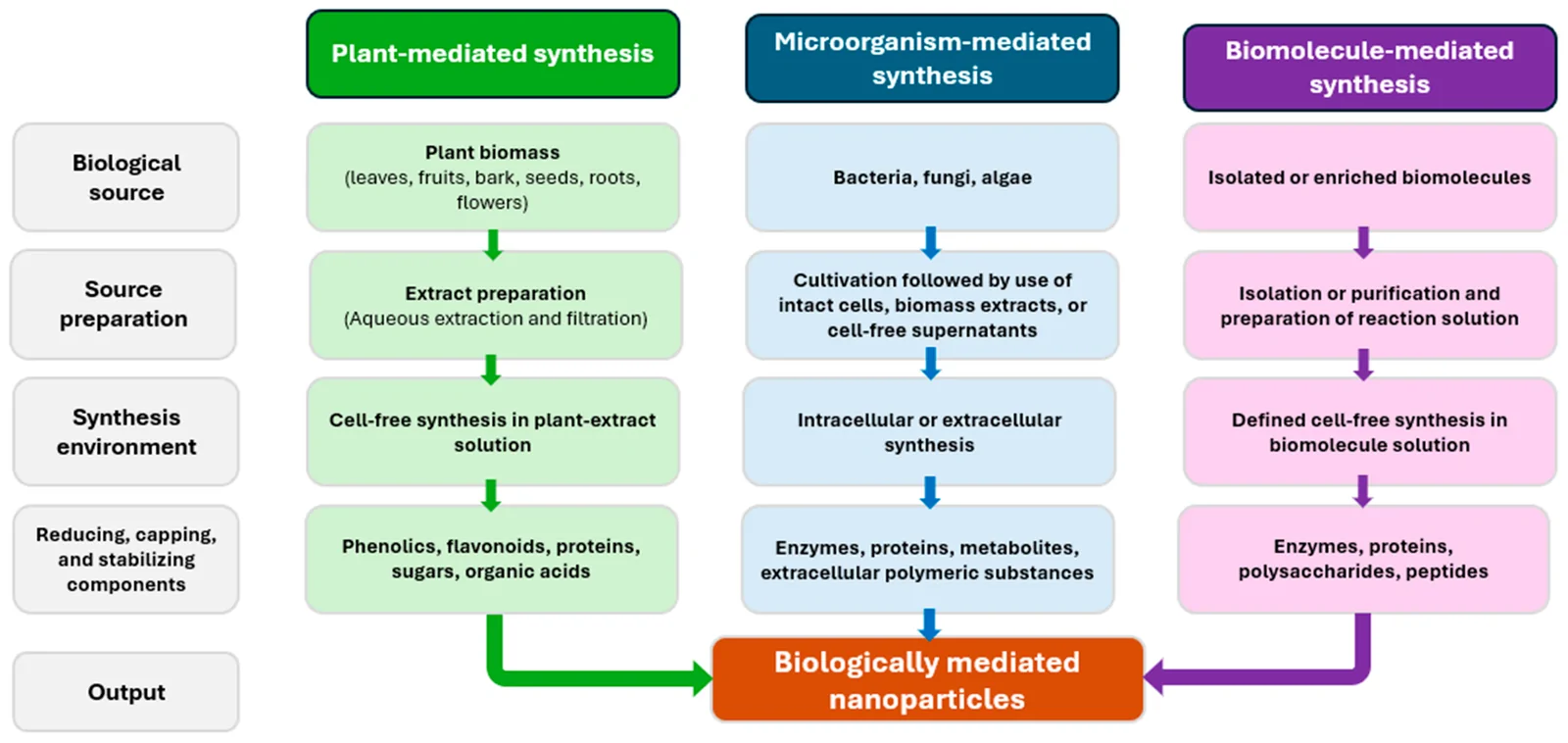
Comparative workflows of plant-, microorganism-, and biomolecule-mediated green nanoparticle synthesis. Plant-mediated routes use phytochemical-rich extracts, microorganism-mediated routes employ intact cells, biomass extracts, or cell-free supernatants, and biomolecule-mediated routes use isolated or enriched biological molecules under defined cell-free conditions. In each route, biological components contribute to metal-ion reduction, nanoparticle capping, and colloidal stabilization.

Biologically mediated nanoparticle synthesis can be classified into three routes: plant-mediated synthesis, microorganism-mediated synthesis, and biomolecule-mediated synthesis. In each route, biological compounds participate in reduction, capping, or stabilization, but differ in operational complexity, compositional control, reproducibility, and potential pharmaceutical suitability [58,78,79].The comparative workflows of these three biologically mediated synthesis routes are summarized in Figure 1. The main characteristics of the three biologically mediated synthesis routes are compared in Table 1.

### 4.1. Plant-Mediated Synthesis

Plant-mediated synthesis uses aqueous plant extracts to generate nanoparticles from metal salt precursors, and naturally occurring phytochemicals may contribute to precursor reduction or transformation, surface capping, and stabilization [58,78,80]. Plant-mediated routes have been applied across metallic and metal-oxide nanoparticle systems, demonstrating their versatility [58,79,80]. These extracts usually contain phenolic compounds, flavonoids, proteins, sugars, and organic acids that participate in metal-ion reduction and capping of the particle surface [58,80,81]. Plant-mediated routes have most often been used for silver and zinc oxide nanoparticles, although the same approach has also been extended to gold, titanium dioxide, selenium, copper, and iron oxide systems [58,82,83]. Plant-mediated synthesis may provide a rapid, simple, and potentially low-cost route for comparative nanoparticle preparation using widely available biomass, with plant extracts supplying reducing and capping biomolecules [78,80,82]. However, its cost-effectiveness and scalability require validation across the complete production process. Differences in plant-extract composition can alter nanoparticle size and morphology, highlighting the need for standardized extraction and synthesis procedures to improve reproducibility and support scale-up [78,84]. Compared with microorganism- and biomolecule-mediated routes, plant-mediated synthesis is usually efficient operationally but offers less precise control over the reducing and capping environment [78,81,85].

### 4.2. Microorganism-Mediated Synthesis

Microorganism-mediated synthesis uses bacteria, fungi, or algae as intact cells, biomass extracts, or cell-free supernatants. In this route, nanoparticle formation often involves enzymatic or metabolism-associated reduction of metal ions, mediated by microbial enzymes, proteins, and secondary metabolites that also contribute to stabilization of the final particles [86,87,88]. Depending on the system, synthesis may occur extracellularly in the culture supernatant or intracellularly within the microbial cell [86,87,89]. Microbial systems have been reported for silver, gold, selenium, and zinc oxide nanoparticles, with some extension to copper oxide systems [86,88,90]. Bacterial systems are often studied in cell-free supernatants or defined cultures, where extracellular enzymes and metabolites support relatively controlled reduction conditions [86,87]. Fungal systems can support extracellular nanoparticle formation because secreted biomolecules, including proteins, enzymes, and secondary metabolites, may contribute to metal-ion reduction, capping, and stabilization [88,91,92,93]. Algal systems provide a distinct aquatic synthesis environment in which polysaccharides, pigments, and other bioactive compounds contribute to reduction and stabilization [90,94]. The main advantages of microorganism-mediated synthesis are the use of defined strains or culture systems and the ability of microbial biomolecules to act as natural reducing and capping agents [86,87,95]. This route is preferred when biological definition, strain-specific synthesis conditions, or intracellular versus extracellular control are important to nanoparticle design. Evidence suggests that algal biomolecules have system-dependent roles: extracellular polymeric substances can support both AgNP reduction and stabilization, whereas components of algal extracts may contribute selectively to either process [96,97]. However, microbial synthesis usually requires culture maintenance, controlled growth conditions, and additional recovery steps, especially when nanoparticles are formed intracellularly [87,89]. Reproducibility may also vary with strain identity, culture age, growth medium, pH, and the use of intact cells, biomass extracts, or cell-free supernatants, because these conditions alter the microbial metabolites and extracellular polymers available for reduction and capping [30,97,98]. Compared with plant-mediated synthesis, microorganism-mediated synthesis can be more operationally demanding because it requires microbial cultivation and controlled growth conditions. However, it may provide a more biologically traceable production system when defined strains or cell-free supernatants are used. Compared with isolated biomolecule-mediated systems, microbial synthesis may still offer less precise control over the final surface environment because complex extracellular biomolecules rather than single defined molecules often mediate reduction and stabilization [85,86,99].

### 4.3. Biomolecule-Mediated Synthesis

Biomolecule-mediated synthesis uses isolated or enriched biological molecules rather than whole plant extracts or living microorganisms. In this route, defined biomolecules act directly in nanoparticle reduction, stabilization, or both. The main biomolecule classes reported in recent primary studies include enzymes, proteins, peptides, and polysaccharides [100,101,102,103]. Enzymes can serve as distinct biocatalysts for metal ion reduction by transferring electrons under controlled reaction conditions, thereby promoting nanoparticle formation through a more specific reduction pathway than crude extract systems [104]. Proteins can act as reducing and capping agents because their functional groups interact with metal ions and nanoparticle surfaces [85,105], whereas polysaccharides such as alginate, konjac glucomannan, and poly-D-mannose are mainly used as stabilizing matrices and may assist nanoparticle formation when combined with reducing biomolecules [106,107,108]. Because these systems are built from defined molecular components, the reduction and capping steps can be tuned more deliberately than in plant- or microorganism-mediated routes. Peptides may also contribute to specific systems [99,107]. The main advantage of biomolecule-mediated synthesis is better control over the reaction environment and surface chemistry compared with crude biological extracts [85,99,107]. This route is especially useful when more defined nanoparticle coatings, improved colloidal stability, or tighter control of surface interactions are required. It is therefore often preferred for systems where precision of capping and interface design matters more than broad exploratory synthesis. Its main limitation is that the number of primary studies is smaller than for plant- and microorganism-mediated synthesis, and the range of nanoparticle types is narrower [85,99,106]. Future studies should test whether isolated enzyme, protein, peptide, or polysaccharide scaffolds require function-specific optimization (reduction, capping, or stabilization) before their extension to additional nanoparticle types [106,107,108]. Compared with plant and microbial routes, biomolecule-mediated systems offer greater molecular definition and control, but they are less broadly used and usually less flexible across source diversity in the current primary literature [85,99,107]. Taken together, these three routes define a practical trade-off within biologically mediated nanoparticle synthesis. Plant-mediated systems are the simplest to scale and screen; microorganism-mediated systems add biological definition and route-specific synthesis environments; and biomolecule-mediated systems provide the greatest control over reduction and surface chemistry. For that reason, comparing them is not only descriptive but also methodological, since the source of reduction and capping directly influences how nanoparticle identity, morphology, surface chemistry, and colloidal stability must be characterized.

## 5. Major Classes of Biologically Mediated Nanoparticles

Biologically mediated synthesis comprises diverse material classes that differ in chemical composition, crystalline structure, surface properties, stability, and biological behavior. These differences influence their synthesis requirements, characterization profiles, mechanisms of action, potential applications, and safety considerations. The following section therefore discusses the major nanoparticle types most frequently reported in biological synthesis studies, including silver, gold, zinc oxide, titanium dioxide, and copper-based nanoparticles.

### 5.1. Silver Nanoparticles (AgNPs)

Silver nanoparticles (AgNPs) are among the most widely investigated biologically mediated nanoparticles. Recent studies indicate that phytochemical components of plants such as flavonoids, phenolic compounds, and terpenoids can act as reducing and stabilizing agents in the synthesis of AgNPs; this phytochemical capping may enhance colloidal stability and modulate the resulting biological activity of the nanoparticles, this phytochemical capping may influence the colloidal stability of the resulting nanoparticles [109,110]. AgNPs synthesized using *Perilla frutescens* leaf extract showed in vitro antibacterial activity against *E. coli* and Staphylococcus aureus, with a minimum inhibitory concentration [110]. Their antimicrobial effects have been associated with nanoparticle-dependent surface reactivity and ROS generation [111]. The same AgNP system showed broad-spectrum antibacterial and biofilm-inhibitory activity together with dose-dependent cytotoxicity and hemolysis, emphasizing the need for concentration-dependent safety assessment [112]. Additional in vitro biological activities, including antioxidant and genotoxic effects, have been reported for specific AgNP formulations [113]. Green-synthesized AgNPs have also shown anticancer activity in experimental models [114].

Moreover, emerging studies have demonstrated the anti-inflammatory activity of AgNPs through modification of cytokine production and reducing levels of oxidative stress [115,116]. AgNPs have also shown inhibition of microbial biofilm development and quorum sensing mechanisms, which enable communication and virulence in bacteria, suggesting considerable potential in combating persistent infections [117]. The role of phytochemically capped nanoparticles has been increasingly recognized in green nanotechnology, with improved dispersibility and stability in aqueous media, which may enhance their interaction with biological systems and improve their suitability for drug-delivery applications [118,119,120]. Further evaluation of toxicity, biodistribution, and long-term safety is required to establish the pharmaceutical suitability of these AgNP formulations [117,121,122].

### 5.2. Gold Nanoparticles (AuNPs)

Green-synthesized gold nanoparticles (AuNPs) have gained increasing attention as biologically active nanomaterials, owing to their chemical stability, functionalizable surface, and relevance to biomedical applications. Recent studies highlight that plant-derived phytochemicals, such as flavonoids, phenolic acids, and terpenoids, act as both reducing and stabilizing agents during AuNP formation. This can reduce reliance on harsh chemical reagents and may enhance the stability and biological functionality of the nanoparticles [123,124]. These nanoparticles, coated with phytochemicals, are better dispersed, less prone to aggregation, and may show altered cellular interaction and colloidal behavior, boosting their therapeutic potential [125]. Moreover, in an in vitro study, the presence of bioactive compounds on the nanoparticle surface further boosts their antioxidant and antimicrobial properties, highlighting the synergistic relationship between the metallic core and the organic coating [126]. An in vitro anticancer study showed that black-tea-extract-mediated gold nanoparticles enhanced doxorubicin-induced cytotoxicity in HCT116 colon cancer cells through a ROS-dependent apoptotic pathway while exhibiting relatively low cytotoxicity toward HEK293 normal kidney cells [127]. This regulated ROS production is particularly crucial to cancer therapy, where AuNPs have been shown to trigger apoptosis through mitochondrial dysfunction, caspase activation, and DNA damage pathways in an oral squamous cell carcinoma study [128]. Additionally, AuNPs, with their large surface area and ease of functionalization, serve as efficient nanocarriers, allowing them to be conjugated with drugs, peptides, and targeting ligands for site-specific delivery and improved therapeutic outcomes [129].

From a physicochemical standpoint, the properties of green-synthesized AuNPs are highly dependent on synthesis conditions, including plant extract composition, pH, temperature, and reaction time. Characterization studies have consistently reported that these nanoparticles are typically spherical, with sizes ranging from 10 to 100 nm, and exhibit strong surface plasmon resonance properties that are crucial for their optical and biological behavior [130]. Recent in vitro and in vivo studies demonstrate that green AuNPs have activity across specific biomedical models. Curcumin–paclitaxel-conjugated biosynthesized AuNPs showed cytotoxic activity against MDA-MB-231 and 4T1 cells and reduced tumor size in 4T1-bearing BALB/c mice [131]. Algal-polysaccharide AuNPs showed antimicrobial activity and inhibited A549, Caco-2, and MCF-7 cancer cells in vitro [132].

In FCA-induced arthritic rats, Manilkara zapota-mediated AuNPs showed antiarthritic effects by reducing paw swelling, joint diameter, TNF-α levels, and oxidative-stress markers [133]. In another study, *Annona muricata*-mediated AuNPs induced dose-dependent apoptosis in SCC-15 tongue squamous carcinoma cells, with increased p53 and Bax expression and reduced Bcl-2 expression [128]. These nanoparticles have also demonstrated antimicrobial activity against tested pathogenic microorganisms, as well as anti-inflammatory effects through modulation of cytokine production and reduction in oxidative stress markers [134]. Biologically mediated AuNPs have also shown wound-healing activity in experimental models [135]. Moreover, the inherent stability and resistance of AuNPs to oxidation make them suitable for long-term biomedical applications, including drug delivery systems and diagnostic platforms [136,137,138]. Overall, green-synthesized AuNPs represent a sustainable and multifunctional nanotechnology platform with significant potential in nanomedicine, supported by a growing body of recent experimental evidence demonstrating their potential in antimicrobial therapy, cancer treatment, wound healing, and targeted drug delivery. Despite these promising findings, challenges remain regarding large-scale production, reproducibility, and comprehensive evaluation of long-term toxicity and biodistribution.

### 5.3. Zinc Oxide Nanoparticles (ZnO NPs)

Zinc oxide nanoparticles (ZnO NPs) are increasingly gaining recognition as an important class of green-synthesized nanoparticles with biomedical relevance, largely because plant-derived metabolites participate in their formation and stabilization. In these systems, plant compounds such as flavonoids, phenolic compounds, and terpenoids not only participate in reduction reactions; they also shape nanoparticle stability, surface chemistry, and interactions with biological systems [139,140]. This biologically derived surface modification improves dispersion in water-based environments, reduces aggregation, and enhances compatibility with cells, ultimately boosting their therapeutic effectiveness [141]. Rather than functioning as passive surface coatings, these phytochemicals actively contribute to the antioxidant and antimicrobial properties of ZnO NPs, creating a synergistic connection between inorganic and organic components [142]. At the same time, ZnO nanoparticles can release Zn^2+^ ions over time in cellular systems, and resulting intracellular zinc accumulation can lead to toxicity through disruption of central metabolic pathways and proteasomal protein degradation [143,144]. This dual mechanism combining oxidative damage with ionic toxicity increases antimicrobial efficacy and may reduce dependence on a single antimicrobial mechanism compared to conventional single-target agents [145,146]. Beyond antimicrobial effects, ZnO NPs demonstrate selective cytotoxicity toward cancerous cells, where elevated ROS levels trigger apoptosis through mitochondrial dysfunction and activation of programmed cell death pathways [147]. Furthermore, their ability to modulate inflammatory responses by regulating cytokine expression and oxidative stress markers supports their use as potential agents in managing inflammatory conditions [148].

From a materials science perspective, synthesizing ZnO NPs using green methods is both simple and adaptable. Typically, plant extracts are combined with zinc precursors such as zinc acetate or zinc nitrate, initiating nanoparticle formation under controlled conditions. Particle sizes are generally reported within the nanoscale range of approximately 10–100 nm, with morphology varying between spherical and hexagonal forms depending on synthesis parameters [139]. These structural characteristics are not only descriptive, but they also directly influence biological interactions, as smaller particles with higher surface area may increase surface reactivity and cellular interaction [149]. In tissue repair contexts, biologically mediated ZnO NPs have also shown wound-healing activity in experimental models [142]. Despite these promising outcomes, it is important to consider the balance between efficacy and safety, as cytotoxic effects remain dose-dependent and require careful optimization.

Although Musa acuminata–mediated ZnO nanoparticles showed lower cytotoxicity than chemically synthesized ZnO nanoparticles in Vero cells, the long-term toxicity, in vivo biodistribution, and clinical translation of green-synthesized ZnO nanoparticle formulations require further investigation [150,151,152]. Overall, ZnO nanoparticles are produced via green-synthesized nanoparticles, integrating materials science and biology to deliver multifunctional therapeutic benefits supported by recent experimental evidence.

### 5.4. Titanium Dioxide Nanoparticles (TiO_2_ NPs)

Green-synthesized titanium dioxide nanoparticles (TiO_2_ NPs) represent other investigated, eco-friendly, and potentially less hazardous alternatives to traditional chemical and physical methods. This synthesis approach uses plant extracts, microorganisms, or biomolecules as reducing and stabilizing agents, which may reduce toxic by-products and energy requirements [153,154,155]. Plant-based synthesis, in particular, has received increasing attention because it can be simple, low-cost, and potentially scalable under standardized conditions. An in vivo study found that *Ocimum sanctum* leaf-extract-mediated TiO_2_ nanoparticles, incorporated into a chitosan gel, improved wound closure in streptozotocin-induced diabetic Wistar rats [156]. These findings support further research on green-synthesized TiO_2_ nanoparticle formulations for topical wound healing. Moreover, using bacteria and fungi in microbial-assisted synthesis introduces enzymatic processes that contribute to nanoparticle formation, often resulting in highly crystalline anatase-phase TiO_2_ with photocatalytic properties [157,158]. These biologically synthesized nanoparticles can show surface-dependent reactivity relevant to applications such as pollutant degradation, antimicrobial activity, and anticancer experimental investigations [159,160,161]. A significant advantage of green-synthesized TiO_2_ nanoparticles is their reported high photocatalytic efficiency under both UV and visible light. This has been attributed to organic residues from biological sources that may change the band gap energy and improve light absorption [162,163,164]. This alteration may lead to a more efficient production of reactive oxygen species (ROS), which are key in degrading organic contaminants and killing microbes. Furthermore, studies point out that these nanoparticles show considerable antibacterial and antifungal activity against a wide array of pathogens [165,166], positioning them as promising candidates for medical coatings [167], wound treatment [156,168], and drug delivery systems [162,169].

Biocompatibility has been reported for some green-synthesized TiO_2_ nanoparticle systems; however, reduced use of hazardous synthesis reagents does not by itself establish lower nanoparticle toxicity or in vivo safety [170]. The structural and functional properties of green-synthesized TiO_2_ can be fine-tuned by adjusting factors like pH, temperature, and extract concentration. This allows for precise control over particle size, crystallinity, and surface area [171]. Additionally, green synthesis methods help improve nanoparticle dispersion and reduce aggregation, which boosts their catalytic efficiency and long-term stability [172]. Another important aspect is the integration of green TiO_2_ nanoparticles into composite materials, where they can be incorporated into polymer or nanomaterial composites or other nanomaterials to enhance mechanical strength, antimicrobial properties, and photocatalytic activity [173].

### 5.5. Copper Nanoparticles (CuNPs)

Green-synthesized copper nanoparticles (CuNPs) are increasingly investigated as alternatives to traditional chemical methods because green routes may reduce hazardous reagents and improve biological compatibility. Copper-based green nanoparticles encompass a broader chemical diversity beyond elemental copper nanoparticles (CuNPs), as recent studies have documented the biological synthesis of copper oxide forms, notably CuO and Cu_2_O nanoparticles [174,175]. Plant extracts from *Aerva javanica*, *Camellia sinensis*, *Prunus africana*, *Ephedra alata*, and *Morinda citrifolia* have been used to synthesize CuO nanoparticles. The resulting nanoparticles have shown antimicrobial, antifungal, antioxidant, photocatalytic, and cytotoxic activities [176,177,178,179]. Similarly, green-synthesized Cu_2_O nanoparticles produced from *Camellia sinensis* and algal extracts have exhibited notable catalytic, antioxidant, and anticancer properties [180,181]. Accordingly, copper-based nanoparticles in this context should be treated as a heterogeneous class encompassing elemental CuNPs, CuO NPs, and Cu_2_O NPs, rather than being confined solely to metallic copper nanoparticles. Green synthesis of copper-based nanoparticles primarily employs plant extracts, microorganisms, and natural biomolecules, which act as both reducing and stabilizing agents [182,183,184]. Recent research indicates that extracts rich in phytochemicals such as flavonoids, phenolics, terpenoids, and alkaloids participate in reducing Cu^2+^ ions and stabilizing nanoscale copper structures. These extracts also reduce aggregation, which may improve colloidal stability and biological activity [185,186]. This method not only reduces reliance on hazardous chemical reagents but also adds surface features that enhance biological activity, making CuNPs particularly appealing for biomedical applications. For example, studies have demonstrated that CuNPs made using plant extracts have antimicrobial activity against both Gram-positive and Gram-negative bacteria. This activity has been associated with reactive oxygen species (ROS) generation, disrupting bacterial membranes, and interfering with intracellular metabolic processes [187]. Additionally, green-synthesized CuNPs have shown antifungal and antiviral activity, expanding their experimental application profile [188,189]. Moreover, factors such as pH, temperature, and the concentration of the extract are crucial in determining the size, shape, and biological activity of the nanoparticles [190]. Smaller, well-dispersed nanoparticles tend to have better surface reactivity and can penetrate biological systems more easily, which boosts their therapeutic potential. In cancer research, green-synthesized CuNPs have demonstrated promising cytotoxic effects against various cancer cell lines. These effects are mediated through oxidative stress, mitochondrial dysfunction, and apoptotic pathways [191]. Importantly, the same study reported that these nanoparticles exhibited comparatively lower cytotoxicity toward tested normal cells in those models, suggesting a level of selectivity that is essential for further safety evaluation. Another major benefit of green-synthesized CuNPs is their antioxidant capacity, largely due to the phytochemicals that remain on their surface. These antioxidant properties enable the nanoparticles to neutralize free radicals and reduce oxidative damage, which could be helpful in managing inflammatory and degenerative diseases [192]. Furthermore, environmentally friendly synthesis routes have been explored using microbial systems like bacteria and fungi, which offer controlled nanoparticle production through enzymatic reduction mechanisms. These biologically synthesized nanoparticles often have unique structural and functional characteristics that differ from those derived from plants, thus expanding their range of applications [192]. Overall, incorporating green chemistry principles into copper-nanoparticle synthesis may reduce hazardous process inputs while influencing nanoparticle surface properties and biological performance.

Table 2 shows that AgNPs currently have the broadest antimicrobial and antibiofilm evidence, whereas AuNPs are particularly relevant to drug delivery, chemosensitization, and diagnostic applications because of their stability and functionalizable surface. ZnO NPs and CuNPs show strong ROS-associated antimicrobial and anticancer potential, but both require careful dose-dependent toxicity evaluation. TiO_2_ NPs are important because of their photocatalytic and antimicrobial properties; however, their discussion should remain focused on biomedical relevance, since environmental and energy applications fall outside the central pharmaceutical scope of this review.

## 6. Characterization Techniques

Characterization in green nanoparticle synthesis is more accurately understood as a convergent workflow rather than a list of separate tests. Across recent primary studies covering plant, microbial, fungal, and biomolecule-mediated systems, spectroscopic, diffraction, microscopic, and colloidal methods were combined because each technique captured a different aspect of nanoparticle identity [78,80,82,85,86,88,95,107]. Various studies demonstrate that no single method is sufficient to establish formation, phase, size, surface chemistry, and dispersion behavior in green-synthesized nanoparticles. This is especially important in biosynthetic systems, where organic coronas, aggregation, and partial amorphous character can complicate interpretation [78,81,95]. The complementary characterization techniques used to evaluate green-synthesized nanoparticles are summarized in Table 3.

### 6.1. UV–Visible Spectroscopy

UV–visible spectroscopy is widely used as an initial indicator of nanoparticle formation because it detects optical changes that emerge as nanoscale products develop in solution. Across recent primary studies, silver nanoparticle absorption maxima were generally reported in the low to mid 400 nm range, including 420 nm in a konjac glucomannan-stabilized system [107], 422 nm in a fungal system [88], 423 nm in a milk protein-stabilized system [85], and 412 to 426 nm across five plant extract-derived preparations [78]. However, one Citrus limon-mediated silver system showed a higher peak at 535.5 nm [80], indicating that peak position varies with particle size, morphology, aggregation state, and surface environment. The same method also distinguished other nanoparticle classes in the current evidence base, with ZnO systems reported near 320 to 355 nm [79,82], selenium nanoparticles at 294 nm [87], and microbial gold nanoparticles at 545 nm [86]. Across these studies, UV-visible analysis consistently supported reduction and early nanoparticle formation, but it could not independently establish crystallinity, distinguish dispersed particles from aggregates, or identify the molecules responsible for capping [78,80,95]. Therefore, UV-Visible results are best considered preliminary evidence of nanoparticle formation and should be confirmed using structural and surface-sensitive characterization techniques.

### 6.2. Fourier Transform Infrared Spectroscopy

FTIR is mainly used to identify which biological functional groups remain associated with the nanoparticle surface after synthesis. Across various studies, the most recurrent signals involved hydroxyl, carbonyl, amine, amide, and carbohydrate-related bands, supporting the view that biological molecules participate in both reduction and capping [80,81,82,85,86,87,107]. In plant-mediated silver and ZnO systems, shifts in O–H, C=O, amide, and C–O bands were consistently interpreted as evidence that polyphenols, proteins, and other metabolites interacted with the nanoparticle surface [78,80,81,82]. Microbial systems showed a similar pattern but with stronger emphasis on proteins and cell wall-associated biomolecules, as reported for *Streptomyces*-derived gold nanoparticles and halophilic bacteria-derived selenium nanoparticles [86,87]. In biomolecule-mediated systems, FTIR interpretation became narrower and more chemically specific. Milk-protein-stabilized silver nanoparticles were linked mainly to amide I and II bands and amino acid side chain interactions [85], while konjac glucomannan-stabilized silver nanoparticles showed a combined polysaccharide and phenolic signature consistent with a more defined stabilizing matrix [107]. The overall pattern is consistent, but the mechanistic precision is limited. FTIR is informative for demonstrating that surface chemistry is modified during synthesis, yet it is less reliable for assigning a unique reduction pathway because many biomolecules produce overlapping bands and several studies reported only broad peak shifts rather than discrete molecular identification [78,82,95]. This ambiguity may be reduced in systems where the reducing or stabilizing components are more clearly assigned, such as milk-protein-assisted AgNPs or konjac-glucomannan-stabilized AgNPs, although these systems are not completely chemically defined [85,107]. FTIR should therefore be treated as supportive evidence for surface association and capping, rather than as stand-alone proof of the exact reducing species.

### 6.3. X-Ray Diffraction

XRD is used to determine whether the synthesized product is crystalline and to identify the dominant phase. Across multiple different studies, silver and gold nanoparticle systems have shown reflections consistent with face-centered cubic metallic structures, including plant, fungal, microbial, and biomolecule-mediated preparations [78,80,85,86,88,107,194]. Plant-based ZnO systems likewise showed characteristic crystalline phases, including wurtzite ZnO in *Aquilegia pubiflora* and phase-pure ZnO matching reference patterns in *Cassia fistula* and Melia azadarach-derived nanoparticles [81,82]. These repeated findings indicate that green synthesis can produce structurally well-defined nanoparticles rather than only poorly organized precipitates.

At the same time, crystallinity is not uniform across green synthesis systems. Selenium-based studies in the same date range show this clearly. One halophilic bacterial system reported an XRD peak consistent with selenium nanoparticle formation [87], whereas a Limosilactobacillus fermentum system showed broad diffraction patterns interpreted as amorphous selenium [95]. In some plant-mediated AgNP preparations, XRD reflections for elemental silver were weak or absent, likely because smaller nanoparticles produced broader diffuse peaks, remained partly dispersed in the supernatant after centrifugation, or generated signals that were lost in background noise [78]. XRD is therefore essential for phase assignment, but its interpretive power declines when crystallites are very small or when organic capping layers mask the signal. In green synthesis studies, this suggests that a weak diffraction pattern should be interpreted cautiously rather than taken as evidence that nanoparticle formation failed.

### 6.4. Scanning Electron Microscopy and Transmission Electron Microscopy

Electron microscopy provides the clearest direct evidence of morphology, but SEM and TEM do not yield equivalent structural information. Across recent primary studies, SEM is mainly employed to assess overall particle morphology, clustering, and surface texture, whereas TEM provides better resolution of individual nanoparticle cores and therefore stronger evidence for nanoscale size [80,82,85,86,88,90,107]. SEM data revealed marked morphological variability across synthesis systems, including spherical particles, clustered rough-surfaced materials, and flower-petal-like CuO structures whose morphology varied with algal extract concentration [82,90]. This suggests that morphology in green synthesis is strongly influenced by extract composition and reaction conditions, not only by the metal precursor.

TEM data showed that core particles were often smaller and more morphologically heterogeneous than SEM imaging alone implied. Plant-mediated silver nanoparticles measured about 10 to 15 nm in a polysaccharide-stabilized system [107] and 7 to 28 nm in a *Citrus limon*-derived system [80] and showed a bimodal distribution with fine spherical nuclei and much larger truncated octahedral particles across different medicinal plant extracts [78]. Microbial gold nanoparticles ranged from 7.1 to 40.0 nm with a mean of 23.2 nm [86], while fungal silver nanoparticles showed quasi-spherical cores around 15 nm [88]. The comparison is important for interpretation: SEM is useful for surveying particle fields and aggregate structure, but TEM is generally the stronger method for core size and shape reporting.

A consistent challenge in nanoparticle characterization is that electron microscopy images may not fully reflect how nanoparticles are dispersed in the original suspension. SEM can exaggerate clustering caused by drying or substrate deposition, while conventional TEM is more useful for assessing primary particle size and morphology but may not capture how particles assemble in bulk suspension. Therefore, SEM and TEM should be considered complementary methods rather than interchangeable ones, with TEM given greater weight for primary particle size and SEM used to provide broader morphological context [85,192,193].

### 6.5. Dynamic Light Scattering

DLS measures hydrodynamic diameter in suspension, so it reflects the particle together with its adsorbed biomolecular layer and associated solvent. Across primary studies, DLS values were consistently larger than microscopy-derived core sizes. *Citrus limon*-mediated silver nanoparticles measured 7 to 28 nm by TEM but 82.51 nm by DLS [80]. Fungal silver nanoparticles measured about 15 nm by TEM but about 47 nm by DLS [88]. In a microbial gold system, TEM gave a mean of 23.2 nm, whereas DLS gave 46.34 nm [86]. A konjac glucomannan-stabilized silver formulation measured 10 to 15 nm by TEM but 60.2 nm by DLS [107], and an Aquilegia pubiflora ZnO system showed 34.23 nm by SEM versus 131 nm by DLS [81]. This repeated discrepancy has a clear interpretation: DLS measures hydrodynamic behavior rather than the physical inorganic core diameter, so larger values are expected when green-synthesized particles carry thick organic coronas or undergo partial aggregation in liquid media [80,86,107]. Accordingly, disagreement between DLS and TEM is not a methodological error. It reflects the fact that the colloidal particle in suspension is not the same physical entity as the dry inorganic core seen by microscopy. The main limitation is that DLS is highly sensitive to aggregation and broad size distributions. In one comparative plant-extract study, photon cross-correlation values substantially exceeded TEM-derived particle diameters, as the technique captured agglomerated grains rather than individual nanoparticles [78]. DLS should therefore not be used in isolation to report particle size. It is substantially enhanced when integrated together with TEM, SEM, or XRD.

### 6.6. Zeta Potential Analysis

Zeta potential analysis is used to estimate surface charge and infer colloidal stability in dispersion. Most green-synthesized nanoparticles carried a negative surface charge, consistent with adsorption of plant metabolites, proteins, polysaccharides, or other biomolecules onto the particle surface [80,81,85,86,87,88,95,107]. Reported values covered a broad range, from weakly negative or near neutral suspensions such as 0.38 mV to −18.1 mV across different medicinal plant silver preparations [78], to more negative systems such as −21.5 mV in Citrus limon silver nanoparticles [80], −26.35 mV in microbial gold nanoparticles [86], −28.8 mV in konjac glucomannan-stabilized silver nanoparticles [107], −32.2 mV in *Limosilactobacillus*-derived selenium nanoparticles [95], and below −50 mV in halophilic bacterial selenium nanoparticles [87]. Across these studies, more negative zeta potential values were generally associated with better colloidal stability, but the evidence also shows that established threshold rules should not be applied too rigidly. Some studies explicitly used a ±30 mV benchmark for stable colloids [95], while others interpreted values around −18 to −26 mV as sufficiently stable in the presence of biological capping layers [81,86]. Near-neutral values were more clearly associated with instability, as seen in the *Berberis vulgaris* silver preparation [78]. Zeta potential is therefore most useful when interpreted together with DLS, visible aggregation, and storage behavior rather than as a stand-alone stability metric. UV–Visible spectroscopy is typically used first to detect the emergence of nanoparticle-associated optical features, FTIR then links the surface to biological capping species, XRD clarifies whether the product is crystalline or amorphous, SEM and TEM resolve morphology and core size, and DLS with zeta potential shows how the same material behaves in suspension [80,82,86,88,95,107]. The main characterization point across these studies is that the most reliable characterization claims come from agreement between methods rather than from any one instrument. A UV–Visible peak can support reduction, but without XRD and microscopy, it cannot distinguish crystalline nanoparticles from aggregated byproducts. FTIR can show that biomolecules remain associated with the surface, but without DLS or zeta potential, it cannot show whether that surface layer stabilizes the dispersion. TEM can confirm nanoscale cores, but without DLS it cannot explain why biosynthesized formulations often appear much larger in suspension [78,81,86]. For this reason, characterization in green nanoparticle synthesis should be presented as a coordinated evidential framework that resolves optical formation, surface chemistry, phase identity, morphology, hydrodynamic behavior, and colloidal stability together.

## 7. Pharmaceutical Applications of Biologically Mediated Nanoparticles

Biologically mediated nanoparticles have been investigated for pharmaceutical applications including drug delivery [195], anticancer therapy [196,197], and antimicrobial applications [198,199], while related diagnostic applications remain at an earlier stage of development [200]. These findings support their investigation as experimental platforms for pharmaceutical applications. These applications depend primarily on the physicochemical and surface properties of nanoparticles. However, their performance is not inherent to the green synthesis approach and may vary according to nanoparticle type, dose, surface chemistry, and biological model [179]. Representative pharmaceutical applications of biologically mediated nanoparticles are summarized in Table 4.

### 7.1. Drug Delivery Systems

Green-synthesized nanoparticles have been investigated for use in drug delivery systems (DDSs) owing to their physicochemical stability, physicochemical characteristics, and surface modification, which can be used to modulate drug pharmacokinetics [201]. In this context, nanoparticle composition, size, surface charge, and biological coating can influence drug encapsulation, cellular uptake, release kinetics, and pharmacokinetic behavior [204,205,206]. Gold nanoparticles (AuNPs) are among the most studied green-synthesized nanoparticles that give promising effects in DDS platforms, as they have been used to modulate doxorubicin (Dox) pharmacokinetics due to their reported biocompatibility, their ability to conjugate with different bioactive molecules, and their size can be adjusted [127,201,202,203]. For instance, AuNPs synthesized using chondroitin sulfate (natural polysaccharide) and chitosan (a natural biodegradable biopolymer) have been reported to sustain doxorubicin release, suggesting a potential strategy for modulating release kinetics and potentially attenuating doxorubicin-associated cardiotoxicity in preclinical models [201,207]. In another study, AuNPs were green synthesized using Peltophorum pterocarpum leaf extract and used as a doxorubicin delivery system. The resulting Dox-loaded AuNPs formulation (b-Au-PP-Dox) exhibited faster cellular uptake and release of Dox compared with unconjugated Dox; this shows the potential of green-synthesized AuNPs to enhance doxorubicin drug delivery and serve as a cost-effective treatment strategy [202]. In addition, the photothermal properties of AuNPs represent the key mechanism for controlling doxorubicin release, as shown by the use of procyanidins, a natural polyphenol, to produce reduced gold nanoparticles coated on doxorubicin-loaded liposomes [203,208]. In an in vitro photothermal drug-release study, laser irradiation of AuNPs generated localized heat that disrupted the liposomal membrane and promoted intracellular drug release [209]. Another study demonstrated the effect of green-synthesized AuNPs from black tea extract in enhancing the chemosensitivity of doxorubicin against colon cancer [127]. Collectively, these studies support the experimental potential of green-synthesized AuNPs as drug delivery platforms, particularly for doxorubicin-based models. Nevertheless, the clinical transition of these systems remains dependent on reproducible synthesis and requires consistent synthesis, efficient drug loading, predictable drug release, stability under physiological conditions, and thorough in vivo safety evaluation.

### 7.2. Anticancer Therapy

Green-synthesized nanoparticles are being investigated in cancer research for their potential cytotoxic effects and ability to enhance therapeutic responses because of the limitations of conventional chemotherapeutic approaches. Green-synthesized AgNPs have demonstrated cytotoxic activity against several cancer cell models, with reported mechanisms including reactive oxygen species (ROS) production, cell-cycle arrest, apoptosis induction, and inhibition of cellular proliferation [214]. In A549 lung-cancer monolayers and multicellular spheroids, *Viridibacillus*-derived AgNPs reduced cell proliferation and DNA synthesis in a concentration-dependent manner. They also caused DNA and mitochondrial damage, mitochondrial depolarization, and apoptosis [210]. In vitro cytotoxicity of green-synthesized AgNPs has been documented in cervical, lung, and breast cancer models; however, these findings arise from separate nanoparticle formulations and experimental systems rather than a single comparative study [210,215,216]. The reported LD50 differed substantially between A549 monolayers and spheroids (1 and 13 μg/mL, respectively), indicating that apparent nanoparticle potency depends strongly on the tumor model used [210]. Black ginger-mediated selenium nanoparticles induced apoptosis and autophagy in AGS gastric cancer cells, accompanied by suppression of PI3K/Akt/mTOR signaling, increased LC3B-II expression, and reduced p62 expression [211]. In the same study, the selenium nanoparticles showed limited cytotoxicity in the tested HaCaT normal keratinocyte model, but this single-cell comparator does not establish general tumor selectivity [211]. Green-synthesized nanoparticles have also been explored as radiosensitizing platforms. For example, green-synthesized gold-coated nanodiamonds have been investigated as potential radiosensitizers for proton therapy [217]. Green-synthesized *Cirsium japonicum*–mediated AuNPs induced iron-dependent ferroptosis in AGS gastric cancer cells. This was associated with mitochondrial ROS production, Fe^2+^ accumulation, lipid peroxidation, and reduced GPX4-related antioxidant activity [212]. At 100–200 μg/mL, these AuNPs reduced AGS-cell viability by 23.7–70.7%, whereas no significant inhibitory effects in RAW264.7 macrophages, normal human dermal fibroblasts, or HEK293 cells at concentrations of 6.25–200 μg/mL, suggesting formulation-specific in vitro selectivity [212]. Chromium oxide nanoparticles provide another example of formulation-dependent activity. *Abutilon indicum*-mediated Cr_2_O_3_ nanoparticles showed cytotoxicity against MCF-7 breast-cancer cells and greater biocompatibility in Vero cells than chemically synthesized Cr_2_O_3_ nanoparticles. However, this evidence remained limited to in vitro experiments [213]. Across these studies, AgNPs were mainly linked to oxidative and mitochondrial damage followed by apoptosis, selenium nanoparticles to apoptosis–autophagy signaling associated with PI3K/Akt/mTOR suppression, and AuNPs to ferroptotic oxidative injury. However, these findings come from separate studies and do not provide direct comparisons of anticancer efficacy between nanoparticle types [210,211,212]. However, anticancer findings remain strongly dependent on nanoparticle type, dose, cancer model, exposure time, and selectivity toward malignant versus normal cells. Most of the available evidence is still based on in vitro studies, with in vivo findings limited to a small number of formulations. In AGS xenograft-bearing mice, Cirsium-mediated AuNPs reduced tumor volume in a dose-dependent manner at 2.5, 5, and 10 mg/kg. Black ginger-mediated selenium nanoparticles also showed antitumor activity in an AGS xenograft model [211,212]. These preclinical findings support further investigation but do not establish clinical efficacy, reproducible biodistribution, or comprehensive long-term safety in normal tissues. Collectively, these findings position green-synthesized nanoparticles as potentially multifunctional oncological agents; however, their differential activity across nanoparticle types, cancer models, and exposure conditions necessitates further systematic investigation before broader conclusions can be drawn.

### 7.3. Antimicrobial Activity

The growing prevalence of antimicrobial resistance has reduced the effectiveness of many conventional antibiotics, increasing interest in alternative antimicrobial strategies. Consequently, attention has turned to the green-synthesized nanoparticles because of their broad-spectrum activity and multiple mechanisms of microbial inhibition [215]. In an in vitro antibacterial study, green-synthesized nanoparticles have shown broad-spectrum antimicrobial activity encompassing Gram-positive and Gram-negative bacteria, fungi, and viruses [218]. Silver nanoparticles are among the most studied green-synthesized nanoparticles against microbial pathogens, including resistant strains, due to their high surface-area-to-volume ratio, ROS generation, membrane disruption, and interaction with microbial proteins or DNA [221,222,223,224,225]. Silver-based materials have a long history of antiseptic use [226] and have been incorporated into pharmaceutical preparations, including ointments and creams, as antiseptics for the management of wounds and burns; all of this establishes a clinically relevant rationale for investigating silver-based nanoparticles as an antimicrobial platform [215]. A recent in vitro study used Mauritia flexuosa fruit extract to synthesize silver oxide nanoparticles and evaluate their antimicrobial activity; the nanoparticles showed antibacterial activity against non-resistant and multidrug-resistant strains, biofilm inhibition, and antifungal activity against *Candida glabrata* [220]. In addition, National Committee for Clinical Laboratory Standards (NCCLS) protocols verified that silver nanoparticles exhibit inhibitory activity against five tested bacterial strains including *Escherichia coli*, *Enterococcus faecalis*, *Pseudomonas aeruginosa*, *Staphylococcus aureus*, and *Streptococcus mutans* [218]. Copper oxide nanoparticles have also shown antibacterial activity against *Bacillus subtilis*, *Escherichia coli*, and *Staphylococcus aureus* and antifungal activity against *Aspergillus flavus*, *Aspergillus niger*, and *Penicillium frequentans* [179]. Antimicrobial activity has also been reported for chromium oxide nanoparticles. For example, Abutilon indicum-mediated Cr_2_O_3_ nanoparticles have antibacterial activity against *E. coli*, *S. aureus*, *B. bronchiseptica*, and *B. subtilis*, showing greater inhibition compared to the plant extracts and commercially synthesized Cr_2_O_3_. This comparison provides evidence that the biological performance of the nanoparticles may differ according to the route of synthesis [213]. Gold nanoparticles also show activity against Gram-positive *Staphylococcus aureus* and Gram-negative *Escherichia coli*, *Klebsiella pneumoniae*, and *Pseudomonas aeruginosa* bacteria [219]. However, antimicrobial activity varies with nanoparticle size, concentration, surface chemistry, organism type, and testing method [46,227,228,229]. These findings suggest that green-synthesized nanoparticles may serve as adjunct or complementary antimicrobial platforms rather than direct replacements for conventional antibiotics.

### 7.4. Diagnostic Contrast-Agent Formulations

Biosensors are analytical platforms used to detect biological or chemical analytes through measurable optical, colorimetric, electrochemical, or fluorescence-based signals [231]. These properties have been used to develop nanoparticle-based sensing systems, where green-synthesized gold nanoparticles have been used to develop highly sensitive colorimetric sensors for the detection of Fe^2+^ [232]. One study used laser ablation in an aqueous gum arabic solution to prepare gold nanoparticles. This biomolecule-assisted method was proposed as a green alternative for producing a potentially cost-effective and biocompatible CT contrast agent [230]. These findings indicate that green-synthesized nanoparticles represent an emerging platform for diagnostic and imaging applications; however, their clinical utility remains contingent upon reproducible synthesis, validated imaging performance, and systematic safety profiling.

## 8. Critical Comparison of Biologically Mediated and Conventional Synthesis

Conventional nanoparticle synthesis commonly relies on physical and chemical methods, which may be associated with several disadvantages, including time-consuming processes, elevated production costs, and high-temperature operating requirements. Chemical methods typically require hazardous reagents, while physical methods demand costly specialized equipment and energy-intensive operating conditions [233,234,235]. Conventional methods may also face problems such as nanoparticle aggregation, cluster formation, relatively large particle size, and instability [236].

Conventionally synthesized nanoparticles have been associated with toxic effects on aquatic organisms, and if these nanoparticles integrate into the food chains, they may pose human health risks through free radical production [236]. Thus, the focus has shifted to using more eco-friendly methods. For example, a comparative cradle-to-gate life-cycle assessment of iron oxide nanoparticles synthesized using *Cymbopogon citratus* extract reported lower normalized environmental impact for the green route than for the conventional coprecipitation method, supporting the environmental advantage of biologically assisted synthesis when process inputs are controlled [237]. Mechanistically, this eco-friendliness is strongest when green synthesis is performed as an aqueous plant-extract process because biological metabolites can replace hazardous synthetic reducing or stabilizing agents while reducing organic-solvent and corrosive-reagent waste [238]. In a comparative LCA of TiO_2_ nanoparticle synthesis, the *Cymbopogon citratus* aqueous-extract route used water and renewable plant-derived reducing/stabilizing compounds, operated at lower temperatures than the chloride route, and showed lower climate-change impact than conventional synthesis (5.27 × 10^−8^ vs. 7.69 × 10^−7^), although ecotoxicity trade-offs from plant-processing residues still required waste control [155]. A 2026 cradle-to-gate LCA of ZnO nanowire synthesis similarly showed that a bio-based glucose/aqueous Fehling-inspired route reduced environmental impacts by one to two order of magnitude across several categories and reduced carbon emissions by 25% compared with sol–gel/chemical bath deposition [239].

There are different methods for the green synthesis of nanoparticles: plant-mediated [240], microorganism-mediated [241], biomolecule-mediated [242], and algae-mediated [243]. This diversity provides flexibility in selecting synthesis routes according to nanoparticle type, intended use, and available biological resources. Green synthesis offers several advantages over conventional approaches, including reduced environmental impact, lower production costs, sustainability, and potential biological compatibility of the resulting nanoparticles [234]. Green synthesis can be cost-effective because it requires milder temperatures, low pressure [236], and less specialized equipment and chemicals [242]. This cost advantage is supported by a 2025 techno-economic and life-cycle analysis of colloidal silver production, in which a plant-extract-based BX3 route—a patented/proprietary plant-extract mixture containing oregano, kale, rosemary, artichoke, and watercress— achieved a higher yield and lower estimated production cost than a sodium borohydride route, although the study also showed that not all green routes are automatically cost-efficient [244]. A 2024 comparative life-cycle assessment of TiO_2_ nanoparticle production found that Cymbopogon citratus extract-based green synthesis achieved a higher yield than the conventional chloride route (92% vs. 74%), with a lower estimated production cost (83.6 vs. 88.88 units) and a slightly shorter reaction time (3 h vs. 3.25 h). These findings suggest potential cost and process-efficiency benefits beyond AgNP systems, although the study also reported possible trade-offs, including higher ecotoxicity linked to plant-production inputs [155]. The use of non-toxic reagents from plant substrates, microorganisms, and biomolecules may improve biocompatibility by reducing exposure to harsh chemical residues [242].

Beyond environmental compatibility, green synthesis offers practical production advantages. Green synthesis supports sustainability by using renewable biological resources. For example, one study used orange peel extract, a natural biowaste material to synthesize magnesium oxide nanoparticles, highlighting the potential of agricultural by-products in nanoparticle production [242,244]. This also frames green synthesis within a circular-bioeconomy model, where low-value agricultural waste is valorized into renewable reducing and capping feedstocks. For example, grape pomace waste and wastewater extracts have been used for water-based, one-pot AuNP synthesis, while tannins extracted from grape pomace have been used to reduce and stabilize AgNPs. These examples show how renewable biological materials could replace conventional synthetic reducing and stabilizing agents [245,246]. In this context, biocompatibility should be distinguished from simple toxicity reduction; it refers to the ability of green-synthesized nanoparticles to interact with biological systems without impairing normal-cell viability, hemocompatibility, or in vivo tolerance at relevant exposure levels [247]. Reduced toxicity is one component of biocompatibility, but biocompatibility should also be demonstrated through positive safety measures such as normal-cell viability or hemocompatibility rather than being assumed simply because acute toxicity is absent [248]. In a 2026 original comparative study of PEGylated ginger gold nanoparticles, green-synthesized AuNPs showed higher neuronal cytocompatibility in PC12 cells than chemically synthesized AuNPs, with >70–80% cell viability at 100 µg/mL and IC_50_ values > 100 µg/mL versus chemically synthesized formulations with IC_50_ values of approximately 68.7–104.4 µg/mL [249]. Improved biocompatibility and reduced toxicity have been reported as advantages of green synthesis in several experimental studies [213,249,250]. Although these outcomes are context-dependent, varying with nanoparticle composition, administered dose, and the biological model employed. In an in vitro experimental study, green-synthesized zinc oxide nanoparticles prepared using *Punica granatum* fruit peel extract showed lower hemolytic toxicity and higher HFF-2 cell viability than chemically synthesized zinc oxide nanoparticles [234]. Lower toxicity has also been reported for green-synthesized AgNPs in vivo. In silver carp, biologically synthesized AgNPs had a higher 96 h LC_50_ value (1300 µg/L) than chemically synthesized AgNPs (700 µg/L) and chemically produced Ag_2_O nanoparticles (50 µg/L). They also caused less disruption in hematological, oxidative-stress, serum-enzyme, and histopathological measures [251]. Similarly, in a *Daphnia magna* acute toxicity model, clove-mediated AgNPs preserved 80–90% survivability across tested concentrations, whereas borohydride AgNPs caused 60–100% mortality, supporting reduced ecotoxicity for this green AgNP system [236]. Together, these findings demonstrate that the advantages of green synthesis are mechanistically linked: biological resources act not only as sustainable raw materials but also as sources of reducing and stabilizing molecules that can influence particle formation, surface chemistry, colloidal behavior, and biological compatibility [234,242,252]. Another comparative study demonstrated that chemically synthesized silver nanoparticles exhibited toxic effects through ROS generation leading to cellular damage and reduced antioxidant capacity, whereas green-synthesized silver nanoparticles showed comparatively lower toxic effects in the same experimental model [240,251].

Collectively, green synthesis may offer environmental, economic, and biological advantages over conventional nanoparticle production, but these advantages should not be presented as universal. Biocompatibility and reduced toxicity remain dose-, nanoparticle-, surface-chemistry-, and model-dependent, as comparative ZnO and AgNP studies show that safety outcomes vary across cell lines, aquatic models, exposure concentrations, and endpoints [234,236,251]. Scalability and batch-to-batch reproducibility also remain major translational barriers, because plant-extract composition can alter nanoparticle synthesis rate, morphology, surface properties, and biological activity [36,253,254,255]. Moreover, not all green routes are automatically cost-efficient or environmentally superior at scale, as techno-economic and LCA studies show that cost and impact depend on yield, biological feedstock processing, waste management, and route-specific inputs [21,237]. Therefore, the advantages of green synthesis require case-by-case validation using standardized biological inputs, controlled reaction parameters, reproducibility testing, and comparative techno-economic or life-cycle assessment before broad generalization.

## 9. Limitations and Challenges

Although biologically mediated nanoparticle synthesis has pharmaceutical potential, its translation remains constrained by biological variability, incomplete process control, scale-up limitations, safety uncertainty, and regulatory requirements.

### 9.1. Biological-Source Variability and Reproducibility

One of the primary limitations is the lack of methodological standardization across synthesis protocols. This lack of standardization can affect nanoparticle pharmacokinetics, biodistribution, and therapeutic efficacy. In biologically mediated synthesis, the presence of heterogeneous phytochemicals or microbial metabolites leads to simultaneous reduction and stabilization processes that are difficult to regulate precisely. For instance, some experimental studies on green-synthesized silver nanoparticles demonstrated that even under controlled experimental conditions, particle size and morphology varied due to differences in plant extract composition [58]. Similar findings have been reported in other nanoparticle types, including zinc oxide and gold, where minimal changes in pH, temperature, or precursor concentration can significantly alter the size and surface properties of the nanoparticles [185,190]. This variability is particularly problematic in nanomedicine, as nanoparticle size governs cellular uptake and toxicity profiles [256,257], tissue penetration [258], and in vivo distribution/retention behavior [259], making precise control essential for drug delivery systems and targeted therapies.

Closely related to the size variability is the issue of poor reproducibility, which represents a crucial barrier to the standardization of biologically mediated nanoparticle synthesis. Unlike many chemical methods that rely on defined reagents and stoichiometric conditions, green synthesis relies on variable factors such as plant species, geographic origin, seasonal variations, and extraction procedures, which can alter the concentration and compositional profile of bioactive compounds involved in nanoparticle reduction and surface capping [260]. Experimental studies on plant-mediated metallic nanoparticles have shown significant variations in nanoparticle yield, morphology, and biological activity from batch to batch due to differences in phytochemical profiles [188,192]. In pharmaceutical development, such inconsistency represents a critical obstacle to producing reproducible pharmaceutical-grade nanoparticles and meeting regulatory quality requirements.

Another central challenge is the limited mechanistic understanding of biologically mediated nanoparticle synthesis which constrains rational process optimization and evidence-based nanoparticle design. Unlike conventional chemical synthesis, where reaction pathways are well-defined, green synthesis involves complex interactions between metal ions and a diverse range of biomolecules, including proteins, enzymes, polysaccharides, and secondary metabolites. Recent studies have indicated that the key mechanisms of nanoparticle formation remain incompletely understood, including their nucleation, growth, and stabilization. These mechanisms may vary according to the biological source used [188,192]. For instance, in plant-mediated synthesis of gold and silver nanoparticles, multiple phytochemicals simultaneously participate in reduction and capping, making it difficult to isolate their individual roles or predict reaction kinetics [84,261]. Similarly, microbial synthesis involves enzymatic pathways that are not fully characterized, further complicating mechanistic modeling [262,263]. A study demonstrated that green-synthesized metallic nanoparticles have complex formation patterns influenced by multiple biochemical factors, highlighting that a unified understanding of their formation mechanism is still lacking [185,190]. This lack of complete understanding hinders the creation of accurate predictive models and makes it difficult to rationally design nanoparticles with specific properties for pharmaceutical and biomedical applications.

### 9.2. Scale-Up and Manufacturing

Another major issue is the challenge of scaling up green synthesis from laboratory-scale experiments to industrial production. While small-scale synthesis using plant extracts or microbial systems is simple and cost-effective, converting these methods to large-scale manufacturing introduces critical technical challenges [253,264]. Studies have reported that parameters such as mixing efficiency, temperature differences, and oxygen availability become increasingly difficult to control in large-scale systems, which can lead to non-uniform nanoparticle formation [185,187]. Furthermore, large-scale production requires a consistent and sustainable supply of biological raw materials, which may be hindered due to environmental and seasonal variability and biological raw material variability, such as plant age, harvest time and storage conditions [260,265,266,267]. For example, microbial synthesis of nanoparticles such as iron oxide and silver faces challenges related to maintaining stable microbial growth conditions and preventing contamination during prolonged production cycles [188]. Additionally, recent studies on green-synthesized metal nanoparticles showed that scaling up led to reduced yield and increased heterogeneity due to mass-transfer limitations and uneven distribution of reducing agents [190]. Therefore, while green synthesis is feasible at the laboratory level, its translation to industrial scale remains constrained by engineering challenges and the ambiguity of the process optimization framework.

### 9.3. Purification, Stability, and Quality Control

Storage stability and shelf-life remain important practical challenges for green nanoparticles. Green-synthesized AgNPs showed gradual physicochemical changes during 18 months of storage, including oxidation/dissolution-related changes and gradual loss of antimicrobial durability. Similarly, green-synthesized AuNPs showed storage-dependent changes in particle size, PDI, and zeta potential over 4–7 weeks [268,269]. These findings suggest that biological or phytochemical capping layers may change over time; therefore, shelf life should be confirmed through formulation-specific stability testing.

Beyond these core issues, biological variability and inconsistent surface chemistry present further challenges for clinical applications. Capping agents derived from biological sources can differ significantly, which leads to variations in surface charge, stability, protein adsorption, cellular uptake, immune interaction, and toxicity [270,271,272,273]. Purification is another challenge for producing pharmaceutical-grade green nanoparticles. Simple methods such as centrifugation, washing, or dialysis may not completely remove unreacted phytochemicals, proteins, polysaccharides, microbial debris, or residual ions. This is relevant because unreacted phytochemicals in green-synthesized ZnO nanoparticles were reported to interfere with MTT and antimicrobial assays unless additional calcination and repeated washing were used. Studies in nanomedicine also show that the purification method itself can affect nanoparticle physicochemical quality attributes [274,275]. Residual biological capping material may also alter surface charge, colloidal stability, protein-corona formation, immune recognition, cellular uptake, and biodistribution, as shown in biologically produced AuNP corona studies and surface-charge-dependent AgNP uptake/biodistribution studies [270,276]. Therefore, green nanoparticles require standardized purification and release criteria distinct from chemically synthesized equivalents, including reporting of purification workflow, residual organic/protein/polysaccharide content, ionic residues, endotoxin or microbial contamination where relevant, and post-purification size, PDI, zeta potential, and storage stability. This variability has been associated with inconsistent toxicity results, where some green-synthesized nanoparticles exhibit selective anticancer activity while others have unintended effects on healthy cells [187,191]. Furthermore, the absence of standardized characterization methods such as size distribution, zeta potential, purification, storage stability, and residual biomolecules makes it difficult to fully evaluate nanoparticle properties.

### 9.4. Safety, Biodistribution, and Protein Corona

The in vitro–in vivo translation gap is another major limitation. Green nanoparticles with complex biological capping layers may behave differently in vivo because protein-corona formation can change their surface identity, targeting ability, cellular uptake, immune recognition, and biodistribution [270,277]. This is particularly relevant for biologically produced AuNPs, as their protein-corona composition differs according to capping agents and plasma concentration, while high-throughput nanoparticle studies suggest that in vitro delivery patterns may not reliably predict in vivo performance [270,278].

A further translational limitation is that in vitro biocompatibility does not necessarily establish in vivo safety. Green-synthesized nanoparticles may still show dose- and exposure-dependent organ accumulation or toxicity after systemic administration; for example, propolis-mediated ultrasmall AuNPs showed higher liver, kidney, and brain accumulation at 100 mg/kg than at 10 mg/kg, while *Ziziphus* leaf-extract AuNPs accumulated proportionally in the liver, spleen, and kidney and were associated with predominant kidney histopathological alterations after acute/chronic exposure [279,280]. Similarly, a 2025 murine study assessed the hematological, biochemical, and histological safety of green-synthesized AgNPs after intravenous administration. However, the 24 h follow-up period was too short to address long-term biodistribution, clearance, immunotoxicity, or repeated-dose safety [281]. In vivo behavior may also be altered by protein corona formation: biologically produced AuNPs showed corona composition changes according to biological capping agents and plasma concentration, while in vivo protein-corona composition has been shown to modify nanoparticle targeting behavior under different disease states [270,277]. Therefore, green synthesis should not be assumed to be systemically safe unless this is supported by long-term studies of biodistribution, clearance, immunotoxicity, hematotoxicity, and repeated-dose toxicity.

### 9.5. Regulatory Barriers

Regulatory translation remains an additional limitation because current FDA and EMA nanotechnology guidance remains product-focused and case-by-case rather than specific to biologically or green-synthesized nanoparticles [282,283,284,285]. FDA guidance regulates nanotechnology products under existing statutory authorities, while EMA states that there is no dedicated EU legal framework for nanomedicines; consequently, green-synthesized nanoparticles may not fit neatly into conventional drug, biological, device, excipient, or combination-product categories, particularly when biological capping layers contribute to stability, biodistribution, or biological activity [286,287,288]. This creates a quality-control problem because batch-to-batch variability in plant- or microbe-derived reducing/capping agents can conflict with ICH Q6A-style expectations for defined specifications, validated test procedures, and justified acceptance criteria for pharmaceutical products [288,289]. Therefore, biologically mediated synthesis translation requires nano-specific regulatory characterization covering critical quality attributes, biological-source variability, residual biomolecules, surface chemistry, stability, sterility, and reproducible manufacturing controls before clinical or pharmaceutical approval can be realistically pursued.

Taken together, while green-synthesized nanoparticles including silver, gold, zinc oxide, iron oxide, and copper nanoparticles are often presented as more sustainable alternatives to traditional nanomaterials, their biomedical and pharmaceutical application remain constrained by challenges associated with particle size control, ensuring reproducibility, achieving scalability, and gaining mechanistic understanding. These limitations are mechanistically linked and stem from the natural complexity and variability of biological synthesis systems. To overcome these challenges, future research must prioritize the development of standardized synthesis and characterization protocols, advanced analytical methodologies, and a more comprehensive mechanistic understanding of nanoparticle formation in biological systems. Addressing these interconnected challenges is a prerequisite for the translation of green nanotechnology from laboratory-scale proof-of-concept investigations into reproducible, regulatory-compliant, and clinically validated biomedical platforms.

## 10. Future Perspectives

Future development of biologically mediated nanoparticle synthesis should move from proof-of-concept synthesis toward reproducible, scalable, and clinically or industrially validated schemes bridging the gap between environmentally driven concepts and robust pharmaceutically reproducible systems. The principal priorities include the standardization of large-scale production, rational development of targeted nanomedicine systems, AI-assisted predictive synthesis design, and systematic safety, regulatory, and translational validation.

### 10.1. Large-Scale Production

Biologically synthesized nanoparticles have emerged as a promising alternative to conventional physical and chemical fabrication industries, offering reduced environmental impact and higher biocompatibility. Green synthesis using plant extracts, algae, fungi, and enzymatic reaction systems relies on safer molecules compared to conventional chemical methods that require hazardous reagents [290]. A major future requirement is the development of scalable and reproducible green synthesis protocols, because variations in experimental conditions can alter nanoparticle size, morphology, surface functionality, and biological performance [291]. Large-scale translation therefore requires moving beyond small-batch protocols toward standardized, process-controlled production platforms. Future manufacturing strategies should also be developed within Good Manufacturing Practice-aligned quality systems by defining critical process parameters, establishing validated production protocols, implementing in-process controls, and setting product-specific batch-release specifications. A recent original study reporting GMP-compliant batch manufacture of dextran-coated iron oxide nanoparticles provides a relevant manufacturing precedent, demonstrating the potential transition of nanoparticle production from laboratory-scale preparation to controlled and reproducible pharmaceutical manufacturing [291]. Although this example was not necessarily based on a green synthesis route, it illustrates the manufacturing controls that future biologically synthesized nanoparticle platforms will need to satisfy before pharmaceutical translation. Recent continuous-flow and bioinspired synthesis studies demonstrate that flow-based green production can improve reproducibility, particle-size control, functionalization consistency, throughput, and scale-up feasibility [292,293,294]. Bioreactor-controlled microbial and fungal synthesis represent a complementary scale-up pathway to plant-based continuous-flow systems. Original studies using cell-free *Streptomyces albus* extract and *Clonostachys rosea* biomass demonstrated that controlled 7 L fed-batch fermentation can increase biosynthesized ZnO and ZnO/MnO nanoparticle yields through regulation of aeration, feeding strategy, and nutrient supply [264,295]. Because microbial extracellular metabolites determine nanoparticle reduction, capping, and stabilization, future bioreactor processes should standardize inoculum quality, dissolved oxygen, pH, feed composition, and harvest time to limit metabolite-related batch variation. Techno-economic and life-cycle assessment data also indicate that future biologically mediated nanoparticle production should evaluate cost, yield, throughput, and environmental impact before industrial adoption [244,296]. Thus, deep mechanistic understanding of how plant-derived biomolecules and fungal compounds mediate nanoparticle formation will be essential for building more rational, predictable, and reproducible synthesis frameworks compatible with standardized large-scale production.

### 10.2. Targeted Nanomedicine

The small size, surface functionality, and potential colloidal stability of green-synthesized nanoparticles make them attractive candidates for drug delivery and targeted nanomedicine applications [201,297,298]. A study was conducted on epigallocatechin gallate (EGCG) to be combined with selenium nanoparticles and starch microgel. Although the development of selenium nanoparticles coated with starch microgel and EGCG extracts is showing great potential as biomedical nanoparticles, this combination has enhanced the bioactivity and anticancer effect through enhancing the anticancer effect against Hep2 cancer cell lines [53]. PFARE-AgNPs were synthesized through an eco-friendly, cost-effective approach and have shown dual anticancer and antibacterial applications. However, further in vivo and in vitro validation studies may be needed [290]. Overall, these green-synthesized nanoparticles show strong anticancer, antibacterial, and antifungal potential, making them promising candidates for future development in novel drug therapies [290]. However, further preclinical studies are required, including dose optimization, biodistribution analysis, pharmacokinetic evaluation, and safety assessment before these are translated into clinical use.

Additional priority is developing stimuli-responsive green nanoparticle delivery systems that release drugs in response to acidic pH, redox imbalance, enzyme activity, or external light. For example, 5-fluorouracil-loaded nanocapsules containing green-synthesized CuO/ZnO nanoparticles released the drug faster at pH 5.6 than at physiological pH, supporting pH-responsive delivery within the acidic tumor microenvironment [299,300]. Similarly, biogenically synthesized Fe_3_O_4_ nanoparticles functionalized with polyethyleneimine and loaded with curcumin exhibited accelerated release at pH 5.2, providing another proof-of-concept for acidic microenvironment-responsive release [301,302]. Photothermal-responsive systems should also be developed further, as green-synthesized apigenin-coated AuNPs and rutin-mediated AuNPs showed laser-dependent photothermal anticancer activity in colorectal and breast cancer cells, respectively [303,304]. Future studies should therefore integrate controlled drug loading with near-infrared-responsive AuNP platforms to enable spatially controlled drug release together with localized photothermal therapy. Passive targeting of systemically administered nanoparticles relies on the enhanced permeability and retention (EPR) effect, whereas active targeting requires post-synthetic surface functionalization with receptor-binding moieties, including antibodies, aptamers, peptides, or small-molecule ligands, to promote target-cell interaction and internalization [305,306]. This approach is compatible with green-synthesized nanoplatforms: plant-derived AuNPs loaded with hypericin and subsequently antibody-conjugated showed enhanced photodynamic cytotoxicity against MCF-7 cells compared with free hypericin in vitro [307]. Similarly, AS1411 aptamer conjugation to flaxseed-mediated AuNPs produced a targeted system with inhibitory activity against MCF-7 cells and minimal effects on normal cells, supporting post-synthetic ligand grafting as a strategy to complement the intrinsic bioactivity of green-derived nanoparticles [308].

Recent in vivo studies provide early support for this translational direction, including green AuNP biodistribution and acute toxicity evaluation [279], biosynthesized AgNP cancer therapy with in vivo bioimaging [247], and green AuNP anticancer activity in liver and breast cancer models [309,310]. However, future targeted nanomedicine studies should move beyond general cytotoxicity by integrating active targeting ligands, controlled drug-loading/release studies, pharmacokinetic profiling, biodistribution mapping, and repeated-dose toxicity assessment before clinical translation can be justified [279,307,311,312]. Chlorogenic acid-rich apple extract served as both a reducing and stabilizing agent in the plant-mediated synthesis of silver nanoparticles (MAgNPs), which exhibited greater antioxidant activity than MLE. Interactions with biomolecules such as DNA, RNA, and proteins may contribute to their reported antiviral, antifungal, and antibacterial activities [40]. Although experimental evidence supports these potential effects, variations in study design, dosing regimens, and comparator conditions require cautious interpretation. Further in vivo studies using standardized protocols are needed [40].

### 10.3. Integration with Artificial Intelligence in Nanoparticle Design

The physicochemical variability in biologically mediated nanoparticle synthesis creates a need for predictive approaches that reduce trial-and-error optimization and improve reproducibility. AI and ML models have been used to relate process variables—including pH, temperature, reaction time, precursor and extract concentrations, feeding rate, and ultrasonic power—to outcomes such as optical absorbance, yield, particle size, zeta potential, morphology, and antibacterial activity [313,314,315,316]. Artificial neural network and response surface methodology (ANN/RSM) models have optimized plant-mediated AuNP synthesis by linking extract volume, precursor concentration, reaction time, pH, and temperature to nanoparticle formation, thereby reducing repeated experiments [313]. ANN was also used to control particle size and improve the antibacterial activity of green cysteine-conjugated AgNPs [313]. Beyond synthesis, nano-QSAR and machine-learning models may help predict nanoparticle toxicity [317,318] and protein-corona composition [319,320]. In the future, digital twins could combine synthesis, toxicity, and nano–bio interaction data to screen formulations before laboratory production, although this approach has not yet been established for green-synthesized nanoparticles [321,322,323]. AI-assisted green nanomaterial design should therefore be developed as a supportive optimization tool for standardized synthesis, scale-up prediction, and preclinical screening, but its outputs still require experimental validation, physicochemical characterization, biological testing, and safety assessment before pharmaceutical or clinical use [314,316].

### 10.4. Safety, Regulation, and Translational Validation

Future translation of green-synthesized nanoparticles requires systematic safety evaluation, toxicological assessment, and regulatory validation, particularly for clinical, pharmaceutical, and food-related applications [234,324,325]. Without standardized safety evaluation criteria, quality-control benchmarks, and dedicated regulatory frameworks for biologically synthesized nanoparticles, their broader industrial and biomedical application will remain limited [326,327,328]. Translation of green-synthesized nanoparticles should not be inferred from successful synthesis alone; recent original studies indicate that biologically produced nanoparticles still require product-specific validation of long-term physicochemical stability, functional durability, cyto-genotoxicity, and organism-level toxicity before they can be justified for biomedical, food-related, or environmental applications [250,268,329,330,331]. Future translation of biologically mediated synthesized nanoparticles should be evaluated through separate validation layers: controlled synthesis and physicochemical quality control [37,58], long-term storage stability [268], in vivo toxicology [117], biodistribution and acute toxicity [279], environmental/ecotoxicological safety [332,333], and preclinical drug-delivery validation [334]. Collectively, these priorities should be approached as a connected translational roadmap rather than as separate development tracks. AI-assisted prediction can support the rational selection and optimization of synthesis conditions, thereby improving batch reproducibility and facilitating scalable, standardized production. Consistent manufacturing and physicochemical control are then required to produce reliable nanoparticle platforms suitable for drug loading, surface functionalization, active targeting, and stimuli-responsive therapeutic delivery. These advanced nanomedicine systems must subsequently undergo structured pharmacokinetic, biodistribution, toxicity, and long-term stability assessment under standardized regulatory frameworks. Progress toward clinical translation will therefore depend on integrating predictive design, scalable synthesis, functional therapeutic development, and regulatory validation within a single quality-by-design pathway.

## 11. Conclusions

Green synthesis should be viewed not only as an eco-friendly method for nanoparticle production but also as a biologically driven approach that can influence nanoparticle properties and function. In this process, biological systems serve as active participants, providing the molecules needed to reduce metal ions and stabilize the resulting nanoparticles. These biological sources should be viewed as design factors because they can shape nanoparticle size, morphology, surface chemistry, colloidal stability, and biological activity. According to the current literature, green-synthesized silver, gold, zinc oxide, titanium dioxide, and copper nanoparticles show relevant experimental activity in antimicrobial, anticancer, drug delivery, biosensing, imaging, and diagnostic models. However, the current evidence for these applications remains largely laboratory-based and should be interpreted with caution. The biological effects of green-synthesized nanoparticles are strongly influenced by particle composition, synthesis conditions, surface coating, dose, exposure time, and the experimental model used. Thus, the current evidence supports their pharmaceutical potential but does not yet justify claims of clinical application. UV–visible spectroscopy, FTIR, XRD, SEM, TEM, DLS, and zeta potential analysis should not be treated as separate descriptive tests but as complementary evidence required to confirm nanoparticle formation, crystallinity, morphology, surface chemistry, hydrodynamic behavior, and colloidal stability. Without this coordinated characterization, biological activity data remain difficult to interpret, compare, or reproduce. Despite their promise, green-synthesized nanoparticles face substantial barriers before pharmaceutical translation. Variability in plant extracts and microbial systems, incomplete control over particle size and surface properties, poor batch-to-batch reproducibility, limited mechanistic understanding, and scale-up difficulties remain unresolved. These problems are not minor technical details; they directly affect safety, pharmacokinetics, biodistribution, efficacy, toxicity, and regulatory acceptability. Overall, green synthesis offers a scientifically valuable route toward more sustainable nanoparticle production, but its future impact will depend on moving beyond proof-of-concept studies. The next stage of the field requires standardized biological-source selection, transparent reporting of synthesis parameters, reproducible characterization workflows, mechanistic studies of reduction and capping processes, and rigorous in vitro, in vivo, toxicological, and pharmacokinetic evaluation. Only through this shift from descriptive synthesis to controlled pharmaceutical development can green-synthesized nanoparticles become reliable candidates for sustainable nanomedicine and biomedical technology.

## Figures and Tables

**Table 1:** Comparative methodological characteristics of biologically mediated nanoparticle synthesis routes.

Biological Route	Biological Source and Synthesis Format	Principal Functional Components and Roles	Reported Nanoparticle Systems	Methodological Strengths and Most Appropriate Applications	Main Limitations and Variables Requiring Control	References
Plant-mediated synthesis	Crude aqueous extracts prepared from botanical materials, including leaves, bark, citrus zest, flowers, and other phytochemical-rich plant tissues	Phenolic compounds, flavonoids, proteins, reducing sugars, organic acids, and related phytochemicals may contribute to metal-ion reduction, nanoparticle-surface capping, and colloidal stabilization	Most commonly AgNPs and ZnO NPs; also reported for AuNPs, TiO_2_ NPs, SeNPs, Cu/CuO NPs, and iron oxide nanoparticles	Rapid preparation, simple handling, relatively low cost, and no requirement for microbial cultivation. Suitable for exploratory synthesis, rapid screening of botanical sources, comparative evaluation of extract-dependent nanoparticle formation, and potential scale-up using widely available biomass	Extract composition may vary with plant species, plant part, geographical origin, maturity, storage, and extraction procedure. Plant identity, plant-part-to-solvent ratio, extraction time and temperature, extract concentration, precursor concentration, pH, reaction time, and reaction temperature should therefore be standardized	[58,78,79,80,81,82,83,84,85]
Microorganism-mediated synthesis	Bacterial, actinobacterial, fungal, or algal cultures used as intact cells, biomass or mycelial extracts, extracellular polymeric substances, or cell-free culture supernatants; nanoparticle formation may occur intracellularly or extracellularly	Enzymes, proteins, secondary metabolites, extracellular polymeric substances, pigments, polysaccharides, and antioxidant compounds may contribute to metal-ion reduction, surface capping, biological-corona formation, and colloidal stabilization	AgNPs, AuNPs, SeNPs, ZnO NPs, and selected CuO NP systems	Enables the use of identifiable strains and controlled culture systems; permits comparison between intracellular and extracellular synthesis; suitable when strain identity, metabolic involvement, biological traceability, or strain-specific synthesis conditions are important	Requires microbial cultivation, contamination control, growth-condition standardization, and downstream nanoparticle recovery. Outcomes may vary with strain identity, inoculum size, growth phase, culture age, medium composition, pH, temperature, biomass concentration, and the use of intact cells, extracts, or cell-free supernatants. Intracellular synthesis may require cell disruption and additional purification	[30,85,86,87,88,89,90,91,92,93,94,95,96,97,98,99]
Biomolecule-mediated synthesis	Cell-free systems containing isolated or enriched enzymes, proteins, peptides, polysaccharides, or biopolymer matrices; individual biomolecules may mediate reduction, capping, stabilization, or combinations of these functions	Enzymes may facilitate electron transfer and metal-ion reduction; proteins may interact with metal ions and nanoparticle surfaces; peptides may direct nanoparticle growth or support surface stabilization; polysaccharides may act as reducing agents, capping agents, or stabilizing matrices	Predominantly AgNPs and AuNPs	Provides greater molecular definition than crude extracts or whole-cell systems and permits more deliberate control over reduction, capping, colloidal stability, and surface chemistry. Suitable for mechanistic studies and systems requiring defined or reproducible surface properties	Biomolecule isolation, purification, and preparation may increase complexity and cost. Performance may depend on biomolecule purity, concentration, molecular weight, conformation, storage conditions, cofactors, additional reductants, and reaction-medium composition. Some systems are hybrid formulations in which different components perform reduction and stabilization	[85,99,100,101,102,103,104,105,106,107,108]

Abbreviations: AgNPs, silver nanoparticles; AuNPs, gold nanoparticles; CuO NPs, copper oxide nanoparticles; NPs, nanoparticles; SeNPs, selenium nanoparticles; TiO_2_ NPs, titanium dioxide nanoparticles; ZnO NPs, zinc oxide nanoparticles.

**Table 2:** Comparative biomedical profile of major, green-synthesized nanoparticle classes.

Nanoparticle Class	Biological Synthesis Context	Key Physicochemical or Surface Characteristics	Representative Biomedical Activities Described	Reported mechanisms in Experimental Models	Main Translational Limitations	References
Silver nanoparticles (AgNPs)	Plant extracts provide constituents that participate in silver-ion reduction, surface capping, and stabilization.	Particle size, surface charge, aggregation, and interactions with proteins depend on the extract and synthesis conditions. Biological capping can influence colloidal behavior.	Antibacterial and biofilm-inhibitory activity in vitro; antioxidant and anti-inflammatory effects in experimental assays; cancer-cell apoptosis; wound-healing activity in rodent models.	ROS accumulation, mitochondrial cytochrome-c release, and caspase activation were demonstrated in selected cervical-cancer cell models. Biofilm inhibition is reported, but its molecular targets are not consistently established.	Toxicity depends on dose, formulation, and exposure conditions. Hemocompatibility, repeated-exposure toxicity, tissue distribution, and batch reproducibility require formulation-specific assessment.	[109,110,111,112,113,114,115,116,117,118,119,120,121,122]
Gold nanoparticles (AuNPs)	Plant extracts or isolated biological constituents support gold-ion reduction and surface stabilization; subsequent conjugation can introduce drugs or targeting groups.	Surface plasmon resonance and a functionalizable surface support optical and delivery applications. Size, morphology, dispersion, and ligand interactions vary between preparations.	Antimicrobial activity; cancer-cell cytotoxicity and chemosensitization in vitro; antitumor effects of drug-conjugated formulations in animal models; anti-inflammatory or antiarthritic effects in selected animal studies.	ROS-dependent enhancement of doxorubicin cytotoxicity and changes in apoptosis-associated proteins or genes have been reported. Drug conjugation provides a delivery strategy whose contribution must be evaluated separately from intrinsic nanoparticle activity.	Results depend on the coating, conjugated drug, dose, and experimental model. Comparative pharmacokinetic, biodistribution, repeated-dose safety, reproducibility, and manufacturing evidence remains limited for the reviewed formulations.	[123,124,125,126,127,128,129,130,131,132,133,134,135,136,137,138]
Zinc oxide nanoparticles (ZnO NPs)	Plant-derived constituents participate in precursor conversion, nanoparticle formation, and surface stabilization.	Particle size, surface charge, aggregation, and interactions with proteins depend on the extract and synthesis conditions. Biological capping can influence colloidal behavior.	Antibacterial activity and cancer-cell viability reduction in vitro; antioxidant activity and a modest fibroblast scratch-closure response; reduced inflammatory markers in a rat infection model.	Zn^2+^ release, oxidative stress, membrane damage, and metabolic disturbances are supported by broader ZnO studies, including non-biological preparations. Their contribution to individual green-synthesized formulations requires direct verification. Reduced cell viability alone does not establish apoptosis or tumor selectivity.	Dose-dependent cytotoxicity and dissolution complicate interpretation. Scratch assays provide preliminary repair-related evidence. Long-term exposure, biodistribution, therapeutic selectivity, and formulation stability require further evaluation.	[139,140,141,142,143,144,145,146,147,148,149,150,151,152]
Titanium dioxide nanoparticles (TiO_2_ NPs)	Plant extracts and microbial preparations can assist precursor transformation and nanoparticle formation; resulting particles may be incorporated into delivery systems or hydrogels.	Crystal phase, particle size, surface composition, aggregation, and light absorption influence performance. Anatase-containing preparations and photocatalytic activity have been reported.	Antibacterial and antifungal activity in vitro; wound-healing effects of nanoparticle-containing formulations in animal models; experimental drug delivery in tumor models; cancer-cell cytotoxicity.	Light-dependent oxidative processes are proposed contributors to antimicrobial effects. Caspase-dependent apoptosis has been reported in a hepatic-cancer cell model. Effects of drug-loaded particles or hydrogels also depend on their other formulation components.	Illumination conditions, crystal phase, and formulation composition affect comparability. Photocatalytic performance does not establish therapeutic efficacy. Repeated-dose safety, systemic distribution, and reproducible pharmaceutical formulation remain insufficiently characterized.	[153,154,155,156,157,158,159,160,161,162,163,164,165,166,167,168,169,170,171,172,173]
Copper-based nanoparticles (Cu, CuO, and Cu_2_O NPs)	Prepared using plant or algal extracts, microbial preparations, or biopolymers. The resulting copper phase must be established experimentally.	Oxidation state, crystalline phase, size, morphology, surface coating, and aggregation determine material behavior. Cu, CuO, and Cu_2_O preparations require separate interpretation.	Antibacterial, antibiofilm, antifungal, and antioxidant activity in laboratory assays; antiviral activity in a specific cell-based system; cancer-cell cytotoxicity and apoptosis in selected formulations.	Bacterial-envelope damage has been observed by microscopy. Apoptosis and cell-cycle changes were reported for algal-derived Cu_2_O nanoparticles in pancreatic-cancer cells. Proposed contributions from ROS and copper-ion release require formulation-specific confirmation.	Chemical and surface heterogeneity restrict comparisons across studies. Copper-related cytotoxicity, changes in oxidation state, colloidal stability, and limited in vivo exposure and safety data complicate translation.	[174,175,176,177,178,179,180,181,182,183,184,185,186,187,188,189,190,191,192]

**Table 3:** Complementary characterization techniques used to evaluate green-synthesized nanoparticles.

Characterization Technique	Primary Output	Interpretation Supported	Key Limitation When Used Alone	Most Informative Complementary Techniques	References
UV–Visible spectroscopy	Wavelength-dependent optical extinction, including localized surface-plasmon resonance bands in suitable metallic nanoparticles and absorption features in semiconductor systems.	Provides preliminary optical evidence consistent with nanoparticle formation and allows monitoring of spectral changes during synthesis or storage.	Biological extracts, residual precursors, and light scattering may contribute to the spectrum. Peak position alone does not establish composition, crystalline phase, particle dimensions, or aggregation state.	Biological-extract and precursor blanks; XRD for crystalline-phase identification; TEM or SEM for morphology; DLS for dispersion behavior.	[78,79,80,82,85,86,87,88,95,107]
Fourier-transform infrared spectroscopy (FTIR)	Vibrational bands and spectral changes associated with hydroxyl, carbonyl, amine, amide, and carbohydrate-related functional groups.	Comparison of adequately purified nanoparticles with the original biological material can support the presence of associated organic constituents and possible capping interactions.	Overlapping bands restrict molecular identification. Residual unbound material may contribute to the spectrum. Band shifts alone do not establish the exact reducing molecule, binding mechanism, coating coverage, or stabilization performance.	Spectra of the original extract or biomolecule, purified nanoparticles, and relevant purification controls; DLS and zeta-potential measurements for dispersion behavior.	[78,80,81,82,85,86,87,95,107]
X-ray diffraction (XRD)	Diffraction-peak positions, intensities, and widths, together with diffuse scattering from poorly ordered material.	Identifies detectable crystalline phases and supports assessment of crystallinity. Appropriate peak-broadening analysis can provide estimates of coherent crystallite dimensions.	Small crystallites, low material content, overlapping reflections, and organic backgrounds may weaken or broaden signals. Absence of sharp peaks does not independently establish an entirely amorphous product. Crystallite size differs from particle size, and minor phases may remain undetected.	TEM or SEM for particle dimensions and morphology; FTIR for organic constituents; comparison with appropriate diffraction reference patterns.	[78,80,81,82,86,87,88,95,107]
Scanning electron microscopy (SEM)	Images of deposited material showing particle or aggregate morphology, surface texture, and dimensions within the instrument’s resolution.	Reveals morphological variation and clustering and permits dimensional measurements when individual particles are adequately resolved.	Drying, substrate deposition, conductive coatings, and image selection can affect apparent morphology and aggregation. Images do not directly represent hydrodynamic dimensions or the original suspension.	TEM for additional assessment of individual particles; DLS for hydrodynamic behavior; examination of multiple representative fields and documentation of sample preparation.	[80,82,85,86,90,192,193]
Transmission electron microscopy (TEM)	High-resolution images of individual particles and aggregates, providing projected dimensions, shape, and an image-based size distribution.	Provides direct evidence of nanoscale particles and supports measurement of primary particle dimensions in the imaged population.	Conventional specimen preparation may introduce drying, aggregation, or sampling artifacts. The imaged population may not represent the entire sample, and low-contrast organic coatings may be poorly resolved. TEM does not directly measure hydrodynamic diameter or long-term dispersion stability.	DLS for suspension measurements; XRD for crystalline-phase information; representative sampling across multiple fields and transparent particle-counting procedures.	[78,80,85,86,88,107,192,193]
Dynamic light scattering (DLS)	Hydrodynamic size inferred from translational diffusion; commonly reported outputs include the cumulants z-average diameter, PDI, and calculated intensity-weighted size distributions.	Characterizes apparent particle size and distribution breadth in a defined liquid medium and can detect changes during aggregation or storage.	Larger particles, dust, and aggregates can disproportionately influence scattering. Results depend on concentration, medium properties, and analysis assumptions. Differences from microscopy may reflect hydration, coatings, aggregation, weighting, or measurement artifacts and do not independently establish coating thickness.	TEM or SEM for particle dimensions and morphology; zeta potential; repeated DLS measurements under defined storage and dispersion conditions.	[78,80,81,86,88,107]
Zeta-potential analysis	Electrokinetic potential at the slipping plane relative to the bulk liquid, inferred from electrophoretic mobility using an appropriate model.	Supports comparison of electrokinetic behavior and the possible contribution of electrostatic repulsion to dispersion stability under defined conditions.	Does not directly measure particle-surface charge or coating composition. Results depend on medium composition, pH, ionic strength, temperature, and conversion model. No universal numerical cutoff establishes stability, particularly when biological coatings provide steric stabilization.	DLS and aggregation measurements over time; storage-stability observations; measurements under matched medium conditions; FTIR as complementary evidence of organic constituents.	[78,80,81,82,85,86,87,88,95,107]

Abbreviations: AgNPs, silver nanoparticles; AuNPs, gold nanoparticles; DLS, dynamic light scattering; FTIR, Fourier-transform infrared spectroscopy; PDI, polydispersity index; SEM, scanning electron microscopy; SeNPs, selenium nanoparticles; TEM, transmission electron microscopy; XRD, X-ray diffraction; ZnO, zinc oxide.

**Table 4:** Representative pharmaceutical applications of biologically mediated nanoparticles.

Pharmaceutical Application	Representative Nanoparticle Systems	Experimental Context	Reported Outcomes	Key Determinants of Activity	Principal Evidence Limitations	References
Drug-delivery systems	Doxorubicin-loaded AuNPs prepared using plant extracts or polysaccharides; chondroitin sulfate–AuNP formulations with chitosan coatings; procyanidin-mediated AuNP-coated liposomes.	Drug-loading and release experiments, cellular uptake and cytotoxicity assays, in ovo assessment, and selected mouse studies.	Formulation-dependent sustained or light-triggered drug release; increased cellular drug delivery and antitumor activity in selected experimental models.	Drug loading, coating composition, particle size, colloidal stability, release conditions, and irradiation parameters for light-responsive formulations.	Results apply to specific formulations. Available animal data do not establish clinical efficacy, general tumor selectivity, or long-term safety. Pharmacokinetics, reproducibility, and stability require further evaluation.	[127,201,202,203,204,205,206,207,208,209]
Experimental anticancer applications and chemosensitization	Microbially synthesized AgNPs; black ginger-derived SeNPs; plant-derived AuNPs and Cr_2_O_3_ nanoparticles; black tea-derived AuNPs administered with doxorubicin.	Predominantly cancer-cell assays, including two-dimensional cultures and three-dimensional spheroids; selected tumor xenograft studies.	Reduced cancer-cell viability; evidence of apoptosis, autophagy-associated responses, or ferroptosis in specific systems; enhanced doxorubicin responses during co-treatment; reduced tumor growth in selected animal models.	Nanoparticle composition, surface chemistry, concentration, exposure duration, cancer model, and treatment combination.	Heterogeneous models and endpoints limit comparison. Mechanisms and selectivity cannot be generalized across nanoparticle classes. Short-term animal observations do not establish comprehensive systemic safety or clinical benefit.	[127,210,211,212,213,214,215,216]
Exploratory radiosensitizer development	Gold-coated nanodiamonds prepared using Nymphaea alba root extract as a reducing agent.	Physicochemical characterization and evaluation of cytotoxicity, cellular uptake, and cell survival in A549 lung cancer cells.	Gold coating and interactions with cells were characterized, supporting further investigation of these hybrid particles as potential radiosensitizers.	Nanodiamond dimensions, thermal pretreatment, gold coating, surface properties, dispersion stability, and cellular uptake.	Material characterization and cellular uptake alone do not establish therapeutic radiosensitization. Radiation-response validation and subsequent in vivo assessment are necessary before therapeutic conclusions.	[217]
Antimicrobial applications	Biologically synthesized AgNPs, silver oxide nanoparticles, CuO nanoparticles, Cr_2_O_3_ nanoparticles, and AuNPs.	In vitro bacterial susceptibility assays, selected fungal assays, biofilm experiments, and evaluation of candidate intracanal antimicrobial materials.	Growth inhibition against tested microorganisms; antibiofilm activity and activity against selected resistant strains in particular formulations.	Nanoparticle composition, size, concentration, surface coating, microbial strain, inoculum, exposure duration, and assay conditions.	Differences in methods and formulations restrict direct comparison. Activity against tested strains does not establish universal broad-spectrum efficacy. Host–cell compatibility, effective exposure at the infection site, and in vivo efficacy remain insufficiently established across these systems.	[46,179,213,215,218,219,220,221,222,223,224,225,226,227,228,229]
Experimental CT contrast agents	Gum arabic-stabilized AuNPs produced by pulsed laser ablation in aqueous media.	CT measurements of nanoparticle-containing agarose phantoms.	Concentration-dependent X-ray attenuation, supporting further investigation as CT contrast materials.	Gold concentration, particle dispersion, coating, and CT acquisition conditions.	Phantom measurements do not establish in vivo diagnostic performance. Standardized comparisons with reference contrast agents, biodistribution, clearance, and toxicity studies are required.	[230]

Abbreviations: AgNPs, silver nanoparticles; AuNPs, gold nanoparticles; Cr_2_O_3_, chromium(III) oxide; CT, computed tomography; CuO, copper(II) oxide; SeNPs, selenium nanoparticles.

## Data Availability

This review is based on the published literature cited in the manuscript. No original datasets were generated.
